# Decreased lncRNA HNF4A-AS1 facilitates resistance to sorafenib-induced ferroptosis of hepatocellular carcinoma by reprogramming lipid metabolism

**DOI:** 10.7150/thno.99197

**Published:** 2024-10-21

**Authors:** Yong Zhao, Shengbo Han, Zhu Zeng, Hai Zheng, Yang Li, Fan Wang, Yan Huang, Yingsong Zhao, Wenfeng Zhuo, Guozheng Lv, Hongda Wang, Guangyu Zhao, Eryang Zhao, Yuhang Hu, Ping Hu, Gang Zhao

**Affiliations:** Department of Emergency Surgery, Union Hospital, Tongji Medical College, Huazhong University of Science and Technology, Wuhan, China.

**Keywords:** hepatocellular carcinoma, sorafenib resistance, ferroptosis, lipid metabolism, m6A, HNF4A-AS1

## Abstract

**Background:** Resistance to sorafenib remains a major challenge in the systemic therapy of liver cancer. However, the involvement of lipid metabolism-related lncRNAs in this process remains unclear.

**Methods:** Different expression levels of lipid metabolism-related lncRNAs in HCC were compared by analysis of Gene Expression Omnibus and The Cancer Genome Atlas databases. The influence of HNF4A-AS1 on sorafenib response was evaluated through analysis of public biobanks, cell cytotoxicity and colony formation assays. The effect of HNF4A-AS1 on sorafenib-induced ferroptosis was measured using lipid peroxidation, glutathione, malondialdehyde, and ROS levels. Furthermore, bioinformatic analyses and lipidomic profiling were conducted to study HNF4A-AS1 involvement in lipid metabolic reprogramming. Mechanistic experiments, including the luciferase reporter assay, RNA pulldown, RNA immunoprecipitation (RIP), methylated RNA immunoprecipitation (MeRIP), and RNA remaining assays, were employed to uncover the downstream targets and regulatory mechanisms of HNF4A-AS1 in sorafenib resistance in HCC. Xenograft and organoid experiments were carried out to assess the impact of HNF4A-AS1 on sorafenib response.

**Results:** Bioinformatics analysis revealed that HNF4A-AS1, a lipid metabolism-related lncRNA, is specifically high-expressed in the normal liver and associated with sorafenib resistance in HCC. We further confirmed that HNF4A-AS1 was downregulated in HCC cells and organoids that resistant to sorafenib. Moreover, both* in vitro* and *in vivo* studies demonstrated that HNF4A-AS1 overexpression reversed sorafenib resistance in HCC cells, which was further enhanced by polyunsaturated fatty acids (PUFA) supplementation. Mechanistically, HNF4A-AS1 interacted with METTL3, leading to m6A modification of DECR1 mRNA, which subsequently decreased DECR1 expression via YTHDF3-dependent mRNA degradation. Consequently, decreased HNF4A-AS1 levels caused DECR1 overexpression, leading to decreased intracellular PUFA content and promoting resistance to sorafenib-induced ferroptosis in HCC.

**Conclusions:** Our results indicated the pivotal role of lipid metabolism-related and liver-specific HNF4A-AS1 in inhibiting sorafenib resistance by promoting ferroptosis and suggesting that HNF4A-AS1 might be a potential target for HCC.

## Introduction

Hepatocellular carcinoma (HCC) is the third leading cause of cancer-related deaths worldwide, accounting for 90% of all primary liver cancers 1,2. Sorafenib, the first FDA-approved molecular-targeted drug for advanced HCC, continues to be a key treatment option in various combinations 3,4. However, the effectiveness of sorafenib is hindered by the inherent or rapid development of acquired resistance within a six-month timeframe. Furthermore, most drugs subsequently tested in phase III clinical trials for advanced HCC have failed to improve upon or achieve efficacy comparable to that of sorafenib 4. Therefore, it is imperative to elucidate the fundamental mechanisms underlying sorafenib resistance to develop novel therapeutic strategies. Reprogramming of lipid metabolism impacts the response to anticancer therapies through various mechanisms, including alterations in plasma membrane or intracellular organelle lipid composition, disruption of the balance between lipid peroxidation and antioxidant defenses 5,6. Aberrant overexpression of sterol-regulatory element binding proteins cleavage-activating protein (SCAP) reduces AMPK activity by increasing cholesterol accumulation and therefore promotes resistance to sorafenib-induced autophagy in HCC 7. Ferroptosis, a form of iron-dependent cell death characterized by excessive accumulation of lipid peroxidation, is closely linked to lipid metabolism 8. In gastric cancer, decreased expression of enzymes ELOVL5 and FADS1 involved in polyunsaturated fatty acid (PUFA) biosynthesis leads to resistance to ferroptosis, which can be reversed by the addition of exogenous PUFA* in vitro* 9. Traditionally known as both autophagy and apoptosis inducer, sorafenib was reported to induce ferroptosis by inhibiting SLC7A11 10. Upregulated unconventional prefoldin RPB5 interactor (URI) in HCC increased the aberrant expression of SCD1, leading to lipid metabolism reprogramming and resistance to sorafenib-induced ferroptosis 11. Given that these studies have revealed the significance of the connection between lipid metabolism reprogramming, ferroptosis and sorafenib resistance, it is imperative to identify the potential roles of lncRNAs in this process.

Several lncRNAs, a type of non-coding RNA larger than 200bp, were documented to mediate sorafenib resistance in HCC. Although lncRNAs may not be the most crucial factors, they remain valuable subjects for research because of their varied regulatory functions. For example, aberrantly overexpressed lncRNA double homeobox A pseudogene 8 (DUXAP8) contributes to sorafenib resistance by promoting SLC7A11 palmitoylation and preventing lysosomal degradation 12. High expression of translation regulatory lncRNA 1 (TRERNA1) caused by HBx promotes resistance to sorafenib by activating the RAS/Raf/MEK/ERK signaling pathway in HCC 13. However, these studies have primarily focused on non-specific lncRNAs, whereas the potential role of relatively liver-specific lncRNAs in mediating distinct functions of the liver, thereby affecting sorafenib resistance, remains largely unexplored. The tissue-type or cell-type specificity of lncRNAs sparks interest in their prospect for targeted and personalized therapies with minimal adverse effects on healthy cells and organs 14-16. Therefore, it is crucial to further investigate the role and therapeutic implications of the relatively liver-specific lncRNAs in sorafenib resistance.

In this study, we analyzed the databases from LIHC of TCGA, GEO (GSE128683, GSE182593), Liver Cancer Model Repository (LIMORE), and Liver Cancer Organoid Biobank (LICOB) and discovered that the lipid metabolism-related and relatively liver-specific lncRNA HNF4A-AS1 is underexpressed in HCC and associated with sorafenib resistance. Furthermore, the results from the parental and resistant cell lines revealed that underexpression of HNF4A-AS1 contributed to sorafenib resistance. We further discovered that HNF4A-AS1 downregulation led to a decrease in intracellular PUFA content according to lipidomic profiling, which consequently suppressed sorafenib-induced ferroptosis. Additionally, we found that HNF4A-AS1 facilitated m6A modification and degradation of DECR1 mRNA by recruiting METTL3 and YTHDF3, as evidenced by RNA pulldown and meRIP assays. Lastly, we observed the synergistic effect of HNF4A-AS1 overexpression and PUFA supplementation in sorafenib treatment of HCC xenograft and organoid models.

## Results

### HNF4A-AS1 is a lipid metabolism-related lncRNA and downregulated in sorafenib resistant HCC cells

**Figure 1:**
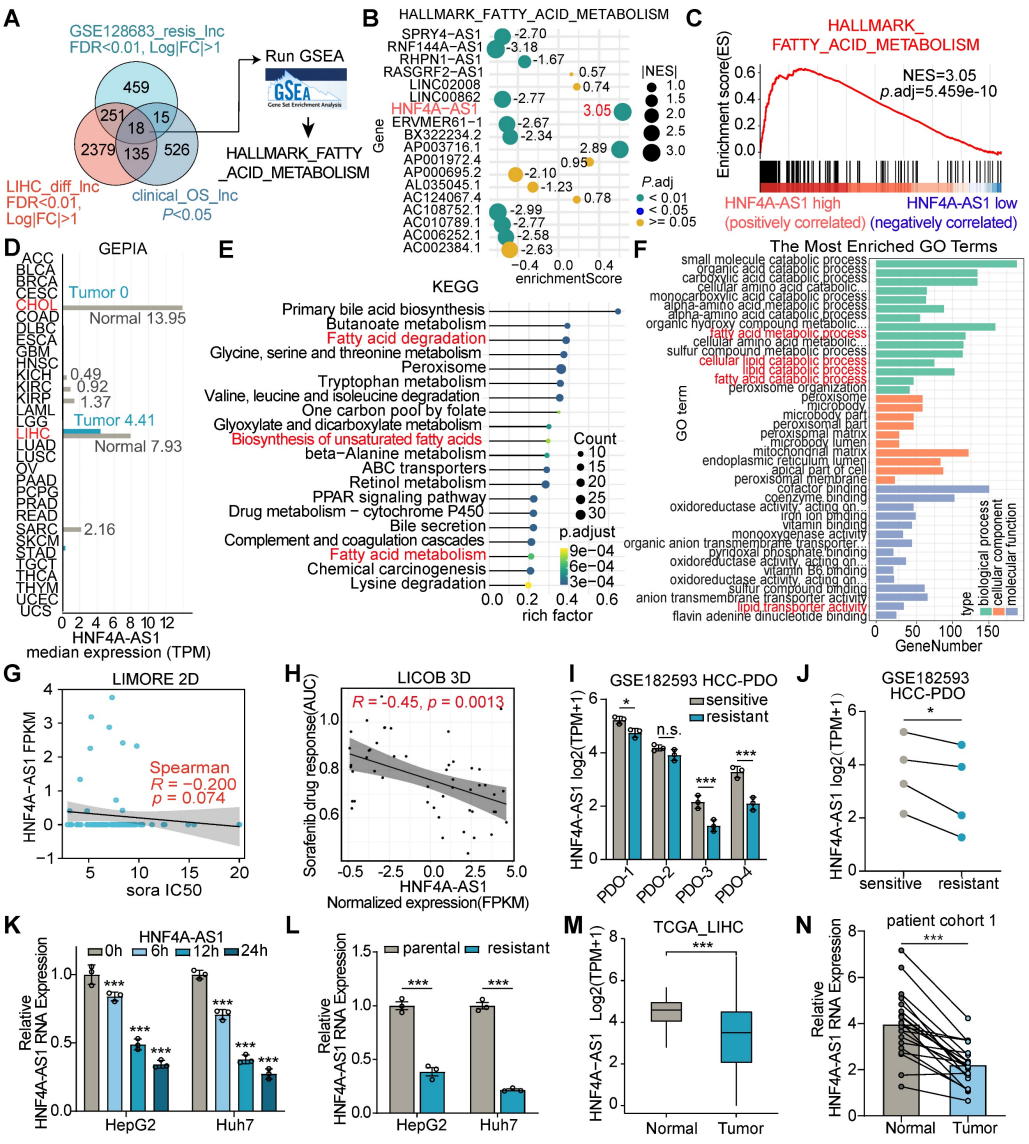
** Lipid metabolism-related lncRNA HNF4A-AS1 is associated with HCC progression and sorafenib resistance.** (A) Venn diagram (left panel) illustrating the identification of differentially expressed lipid metabolism-related lncRNA that both exhibit relevance to sorafenib resistance (GSE128683) and have significant overall survival (OS) implications in the TCGA-LIHC datasets. The right panel showing the identification of fatty acid metabolism-related lncRNAs by GSEA analyses. (B) Bubble plot showing the GSEA analyses of 18 lncRNAs using fatty acid metabolism gene set in the TCGA database. The numerical annotations in the figure represent NES (normalized enrichment score). (C) GSEA analysis of HNF4A-AS1 in LIHC. NES: normalized enrichment score. (D) Expression levels of HNF4A-AS1 across various cancer types in the GEPIA database (http://gepia.cancer-pku.cn/). (E-F) KEGG and GO analyses of HNF4A-AS1 in LIHC. (G-H) The relationship between sorafenib response and HNF4A-AS1 expression was observed in both primary liver cancer cells cultured in 2D (LIMORE, www.picb.ac.cn/limore) and organoids cultured in 3D (LICOB, www.cancerdiversity.asia/LICOB) from the respective databases. Higher IC50 and AUC indicate lower sorafenib response. (I-J) GSE182593 dataset displaying the expression levels of HNF4A-AS1 in 4 paired sensitive and resistant HCC PDOs. (K) Expression levels of HNF4A-AS1 in HepG2 and Huh7 cells before and after treatment with sorafenib at different time gradients. (L) Expression levels of HNF4A-AS1 in parental and resistant HepG2 and Huh7 cells. (M) Expression levels of HNF4A-AS1 in tumor and normal tissues according to TCGA LIHC database. (N) Relative expression levels of HNF4A-AS1 were determined in specimens of untreated HCC patients using RT-qPCR. N = 20. CHOL: cholangio carcinoma. LIHC: liver hepatocellular carcinoma. sora: sorafenib. PDO: patient-derived organoid. Data shown are means ± SD from biological triplicates. **:*P* < 0.01, ***:*P* < 0.001, n.s.: *P* > 0.05.

To explore the potential lipid metabolism-related lncRNAs associated with sorafenib resistance in HCC, we integratively analyzed datasets from the sorafenib-resistant HepG2 database GSE128683, the Liver Hepatocellular Carcinoma (LIHC) derived from The Cancer Genome Atlas (TCGA) database, and overall survival associated lncRNAs. We identified 18 lncRNAs that were significantly associated with the sorafenib resistance in hepG2 cell line, and differentially expressed in HCC patients based on the clinical progression (**Figure 1**A). Then, gene set enrichment analysis (GSEA) was performed to discover fatty acid metabolism-related lncRNAs (Figure 1B). Notably, HNF4A-AS1, which is highly expressed specifically in the normal liver, emerged from our findings (Figure 1C-D). The GEPIA database exhibited dramatically higher expression levels in normal liver tissues, including cholangial carcinoma (CHOL) and liver hepatocellular carcinoma (LIHC), than in other tissues (Figure 1D). The Genotype-Tissue Expression (GTEx) database, derived from healthy human individuals, reflectes its relatively specific expression pattern. HNF4A-AS1 expression in the healthy liver (median TPM = 11.16) was markedly higher than in other healthy tissues, including the small intestine (median TPM = 2.063), kidney (median TPM = 1.162), stomach (median TPM = 0.00), and brain (median TPM = 0.00) (Figure S1A). Subsequently, we validated the organ specificity of HNF4A-AS1 in various tumor cells, revealing its exclusive expression in liver cancer cells, whereas it was absent in tumor cells from other organs (Figure S1B). Therefore, we propose that HNF4A-AS1 exhibits a relatively liver-specific expression pattern. Additionally, the results from multiple liver-derived cell lines showed that HNF4A-AS1 expression in normal MIHA cell lines significantly surpassed that in multiple HCC cell lines (Figure S1C), reaffirming the observed reduction in GEPIA (Figure 1D).

Moreover, Kyoto Encyclopedia of Genes and Genomes (KEGG) pathway analysis indicated that HNF4A-AS1 was significantly associated with fatty acid degradation and the biosynthesis of unsaturated fatty acids (UFA) (Figure 1E). Gene ontology (GO) analysis based on the LIHC database revealed that HNF4A-AS1 is involved in various lipid metabolism pathways, including fatty acid metabolism, lipid catabolism, and lipid transporter activity (Figure 1F).

To assess the influence of HNF4A-AS1 on sorafenib treatment, we first predicted the correlation between HNF4A-AS1 and sorafenib response in the Liver Cancer Model Repository (LIMORE) and the Liver Cancer Organoid Biobank (LICOB) 17,18. The results indicated that lower expression of HNF4A-AS1 was associated with a reduced response to sorafenib (higher IC50 and AUC) (Figure 1G-H). The RNA-seq of HCC patient-derived organoids (GSE182593), which were established to paired sorafenib-sensitive and -resistant organoids, also revealed lower expression of HNF4A-AS1 in the resistant organoids compared to the paired sensitive organoids (Figure 1I-J). Next, we investigated the effect of sorafenib treatment on HNF4A-AS1 expression using a time-gradient approach. The results indicated a gradual decrease in the expression of HNF4A-AS1 with increasing treatment duration (Figure 1K). Subsequently, we developed sorafenib-resistant cell lines HepG2-SR and Huh7-SR and confirmed the resistance status using the CCK-8 assay. Both HepG2-SR and Huh7-SR cells showed enhanced resistance to sorafenib compared to their parental counterparts (Figure S1D-E). RT-qPCR analysis also revealed a lower expression of HNF4A-AS1 in resistant cells than in parental cells (Figure 1L).

Furthermore, we discovered that HNF4A-AS1 expression was downregulated in HCC, and negatively correlated with advanced clinical pathological stages and elevated AFP expression (Figure 1M, and Figure S1F-G). Meanwhile, we examined the expression of HNF4A-AS1 in cohort 1 of untreated patients, consisting of 20 individuals. The results also revealed that the expression of HNF4A-AS1 was lower in HCC tissues than in the adjacent normal tissues (Figure 1N). In continuation, we investigated the expression of HNF4A-AS1 using the gene expression profile in liver cells and tissue (GepLiver) database, an integrative liver expression atlas that covers developmental stages and phases of liver disease 19. We observed aberrant expression of HNF4A-AS1 in various liver diseases, with elevated expression in NAFLD and cirrhosis, and decreased expression in HCC (Figure S1H). Moreover, survival analysis confirmed that lower HNF4A-AS1 expression was associated with poorer overall survival (OS) and disease-free survival (DFS) in the LIHC database (Figure S1I-J).

Next, the CPC2 and PhyloCSF databases were used to predict the protein-coding ability of HNF4A-AS1, and the results confirmed that HNF4A-AS1 is a *bona fide* non-coding RNA (Figure S1K). The RNAfold webserver was used to predict the secondary structure of HNF4A-AS1 (Figure S1L).

In summary, we discovered that the relatively liver-specific lncRNA HNF4A-AS1 is downregulated in HCC and is associated with poorer prognosis.

### Hypoxia reduced HNF4A-AS1 expression via HIF-1α

**Figure 2:**
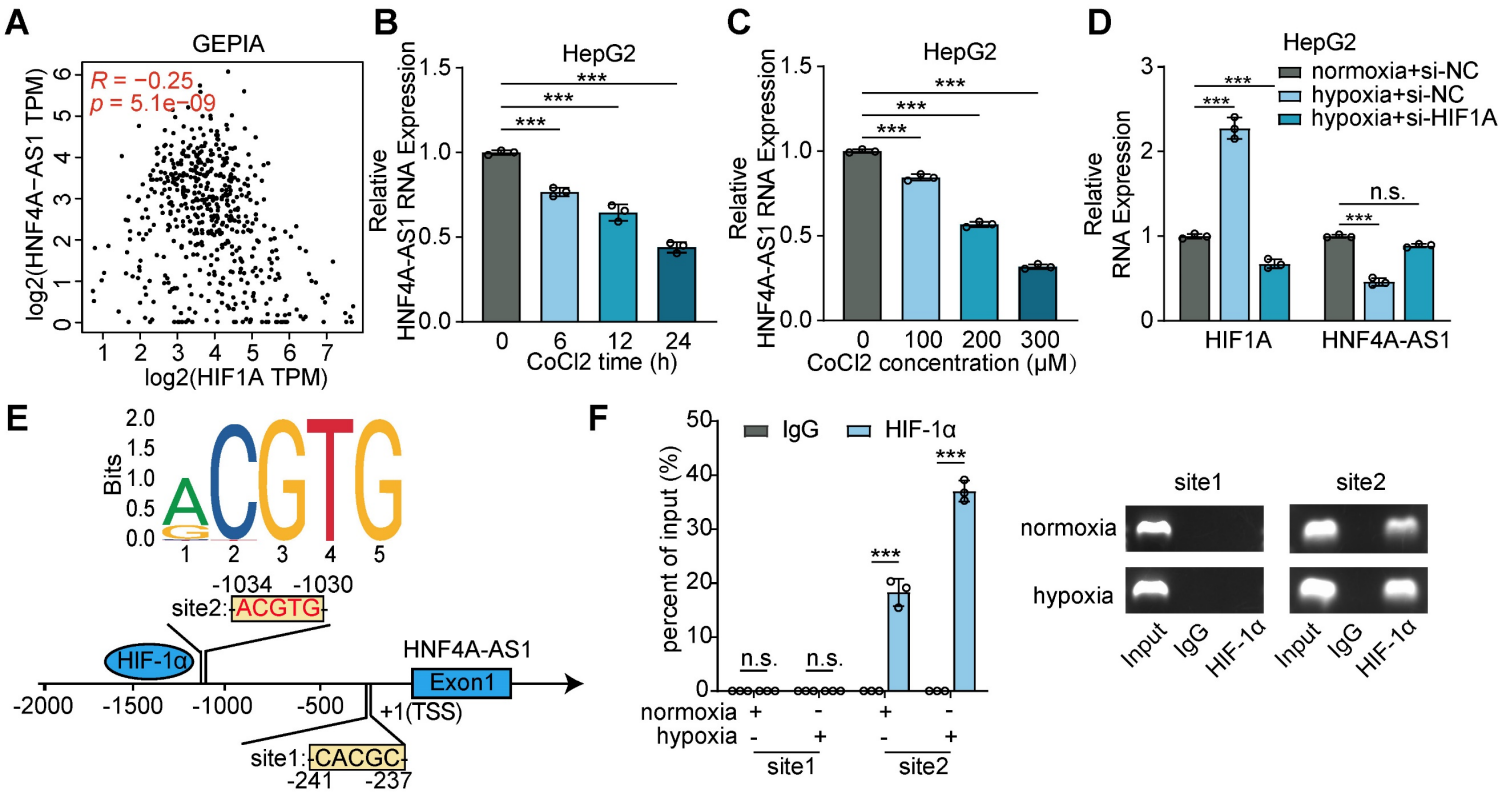
** Hypoxia downregulated HNF4A-AS1 via HIF-1α.** (A) Correlation between HIF1A and HNF4A-AS1 RNA expression of LIHC in GEPIA was analyzed by Pearson's correlation. (B-C) The expression of HNF4A-AS1 under different treatment duration and concentration of CoCl2 in HepG2 were determined by RT-qPCR. (D) The expression of HIF1A and HNF4A-AS1 in HepG2 cells cultured under normoxia or hypoxia and co-transfected with siNC or siHIF-1α were assessed by RT-qPCR. (E) Schematic diagram of the potential binding sites for HIF-1α in the promoter region of HNF4A-AS1 using JASPAR database. (F) ChIP-PCR assay with HIF-1α antibody or IgG was conducted to explore the binding between HIF-1α protein and binding sites of HNF4A-AS1 promoter in HepG2 cells under normoxia and hypoxia, followed by visualization on 2% agarose gel. Data shown are means ± SD from biological triplicates. ***:*P* < 0.001, n.s.: *P* > 0.05.

Subsequently, we aimed to elucidate the mechanism underlying the downregulation of HNF4A-AS1 expression. Previous studies have established a relationship between hypoxia, HIF-1α, and sorafenib resistance in HCC 20,21. Intriguingly, our analysis of the GEPIA database revealed a positive correlation between HIF-1α and HNF4A-AS1 expression (**Figure 2**A). CoCl2 is frequently employed as a simulation model for hypoxic conditions, because it induces chemical hypoxia and stabilizes hypoxia-inducible factors 1α and 2α even under normoxic conditions 22. The results of the CoCl2 treatment assays demonstrated that both the duration and concentration of CoCl2 exposure significantly inhibited HNF4A-AS1 expression (Figure 2B-C). Moreover, we performed hypoxia treatment with si-HIF-1α knockdown to investigate the effect of HIF-1α on HNF4A-AS1. The PCR results showed that hypoxia induced HIF-1α expression and downregulated HNF4A-AS1 expression. However, si-HIF-1α knockdown rescued the hypoxia-induced overexpression of HIF-1α and the downregulation of HNF4A-AS1 (Figure 2D). To explore whether HIF-1α transcriptionally represses HNF4A-AS1, we predicted the HNF4A-AS1 promoter sequence motif (MA0259.2) using the JASPAR database (Figure 2E). The ChIP-PCR assay revealed that only the region at -1030 to -1034 bp upstream of the HNF4A-AS1 transcription start site (TSS) could bind to HIF-1α (Figure 2F).

In summary, our findings indicated that hypoxia suppresses the transcription of HNF4A-AS1 via HIF-1α.

### Decreased expression of HNF4A-AS1 contributes to sorafenib resistance in HCC cells

**Figure 3:**
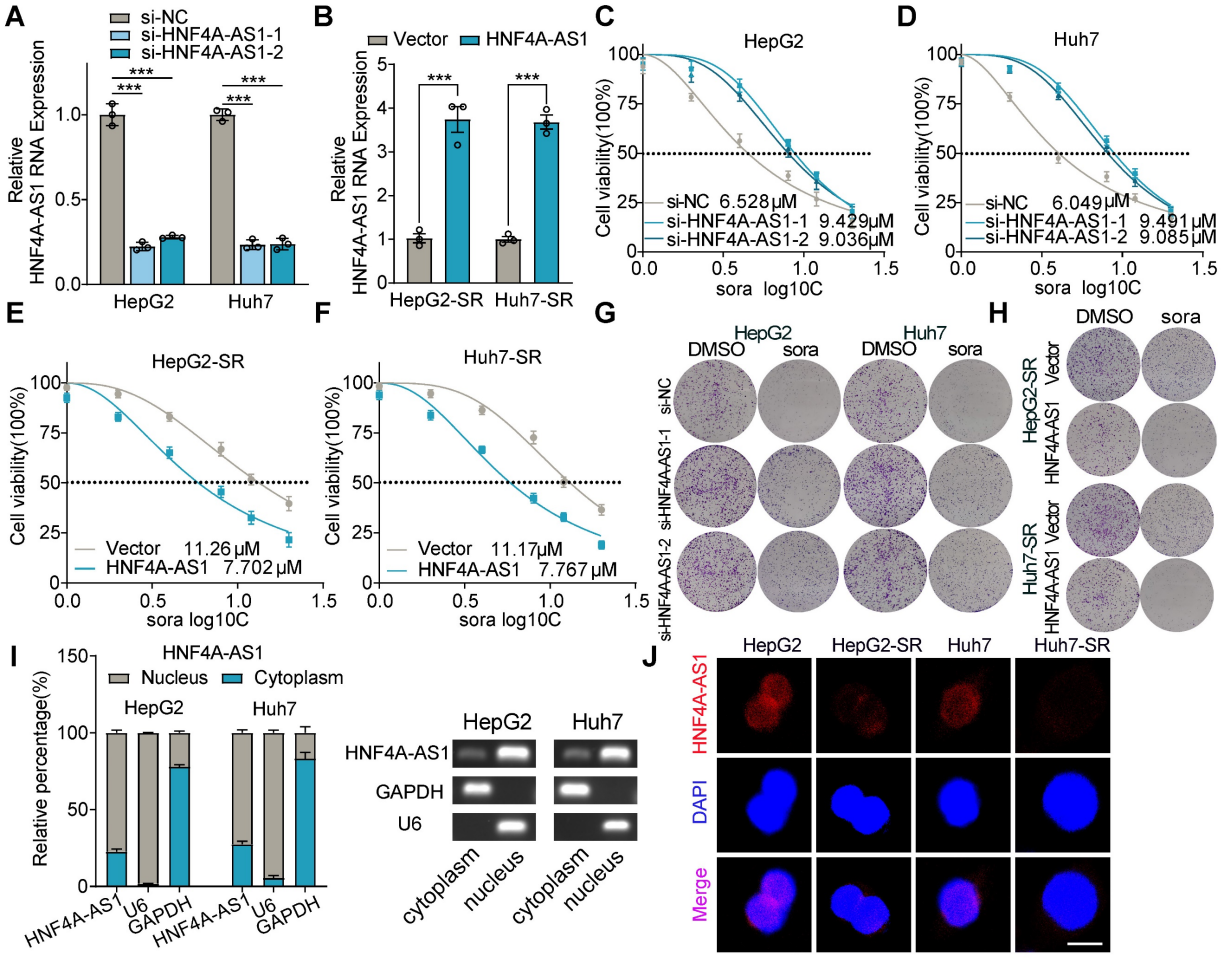
**Downregulation of HNF4A-AS1 leads to sorafenib resistance.** (A-B) The efficiency of interfering with si-HNF4A-AS1 in the HepG2 and Huh7 cells and overexpressing HNF4A-AS1 in the HepG2-SR and Huh7-SR cells was determined by RT-qPCR. (C-D) Sorafenib IC50 values in HepG2 and Huh7 cells after interference of HNF4A-AS1 expression. (E-F) Sorafenib IC50 values in HepG2-SR and Huh7-SR cells after overexpression of HNF4A-AS1. (G) Colony formation assay testing the response to sorafenib (10 μM) in HepG2 and Huh7 cells after interference of HNF4A-AS1 expression. (H) Colony formation assay testing the response to sorafenib (10 μM) in HepG2-SR and Huh7-SR cells after overexpression of HNF4A-AS1. (I) Subcellular fractionation and RT-qPCR analysis detected the subcellular expression of HNF4A-AS1 in HepG2 and Huh7 cells. (J) RNA-FISH assay of HNF4A-AS1 in paired parental and resistant HepG2 and Huh7 cells. HepG2-SR: sorafenib-resistant hepG2. Huh7-SR: sorafenib-resistant huh7. Sora: sorafenib. Scale bar:10 μm. Data shown are means ± SD from biological triplicates. *:*P* < 0.05, ***:*P* < 0.001, n.s.: *P* > 0.05.

To determine the effect of HNF4A-AS1 on sorafenib resistance, we conducted gain- and loss-of-function assays. HNF4A-AS1 was manipulated with siRNA (si-HNF4A-AS1-1/2) in parental cells and overexpression plasmids in resistant cells, respectively (**Figure 3**A-B). Downregulation of HNF4A-AS1 in parental cells led to increased sorafenib resistance (Figure 3C-D), as demonstrated by the IC50 assay, whereas overexpression in resistant cells enhanced sensitivity to sorafenib (Figure 3E-F). Concordantly, the colony formation assay confirmed that reduced expression of HNF4A-AS1 diminished the sorafenib response, whereas overexpression of HNF4A-AS1 enhanced the response to sorafenib (Figure 3G-H). Furthermore, we investigated the impact of HNF4A-AS1 on the response to two clinically utilized tyrosine kinase inhibitors (TKIs), regorafenib and lenvatinib. Analysis using the LIMORE and LICOB databases revealed a negative association between HNF4A-AS1 expression and regorafenib response in HCC (Figure S2A-B). Subsequent IC50 assay results indicated that HNF4A-AS1 knockdown impeded the response of HepG2 to regorafenib (Figure S2C), while HNF4A-AS1 overexpression enhanced sensitivity to regorafenib (Figure S2D). However, HNF4A-AS1 did not impact the sensitivity of HCC cells on lenvatinib, as demonstrated by analysis from the LIMORE, LICOB and IC50 assays (Figure S2E-H).

Subcellular fractionation assays by RT-qPCR showed that HNF4A-AS1 was mainly distributed in the nucleus, and fluorescence *in situ* hybridization (FISH) assay also confirmed this (Figure 3I-J). In addition, FISH assay also demonstrated that HNF4A-AS1 was downregulated in paired sorafenib resistant HCC cells (Figure 3J).

Collectively, these results indicated that HNF4A-AS1 downregulation contributes to the development of sorafenib resistance.

### HNF4A-AS1 regulates sorafenib sensitivity by ferroptosis in HCC cells

**Figure 4:**
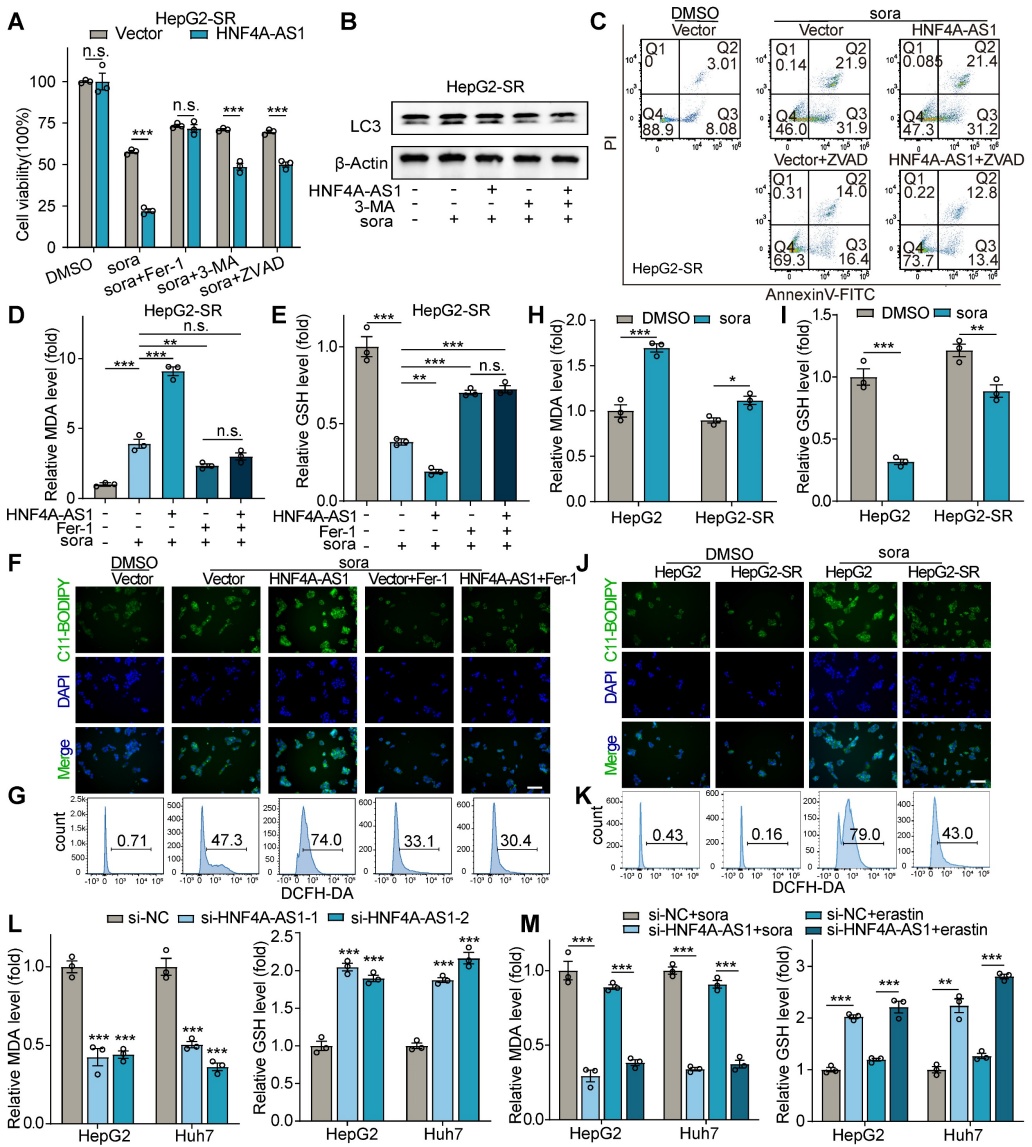
**HNF4A-AS1 regulates sorafenib response through ferroptosis.** (A) Cell viability assay of the effects of ferroptosis inhibitor ferrostatin-1 (Fer-1, 10 μM), autophagy inhibitor 3-methyladenine (3-MA, 10 μM) or apoptosis inhibitor Z-VAD-FMK (ZVAD, 50 μM) on cell viability after sorafenib treatment (10 μM) in HepG2-SR cells overexpressing HNF4A-AS1 or Vector. (B) Western blot demonstrating the impact of HNF4A-AS1 overexpression and autophagy inhibitor 3-MA (10 μM) on autophagy in HepG2-SR cells after sorafenib treatment (10 μM). (C) AnnexinV/PI flow cytometry experiment revealing the influence of HNF4A-AS1 overexpression and apoptosis inhibitor ZVAD (50 μM) on apoptosis in HepG2-SR cells after sorafenib treatment (10 μM). (D-G) MDA assay (D), GSH assay (E), C11-BODIPY labeled LPO (F), and DCFH-DA labeled ROS (G) demonstrating the effects of HNF4A-AS1 overexpression and ferroptosis inhibitor Fer-1 (10 μM) on induced ferroptosis in HepG2-SR cells after sorafenib treatment (10 μM). Scale bar:50 μm. (H-K) MDA assay (H), GSH assay (I), C11-BODIPY labeled LPO (J), and DCFH-DA labeled ROS (K) showing that ferroptosis is less prominent in HepG2-SR cells compared to HepG2 cells under 10 μM sorafenib treatment. Scale bar:50 μm. (L) MDA and GSH assay indicating that si-HNF4A-AS1 reduces ferroptosis in HepG2 and Huh7 cells under sorafenib treatment (10 μM). (M) MDA and GSH assay illustrating that si-HNF4A-AS1 also decreases ferroptosis caused by erastin (10 μM) in HepG2 and Huh7 cells. 3-MA: 3-methyladenine. Fer-1: ferrostatin-1. HepG2-SR: sorafenib-resistant hepG2. Huh7-SR: sorafenib-resistant huh7. Sora: sorafenib. ZVAD: Z-VAD-FMK. Data shown are means ± SD from biological triplicates. *:*P* < 0.05, **:*P* < 0.01, ***:*P* < 0.001, n.s.: *P* > 0.05.

Sorafenib, commonly recognized for its roles in inducing autophagy and apoptosis, has recently been identified as an inducer of ferroptosis 10. Therefore, we investigated the mechanism by which HNF4A-AS1 regulates sorafenib resistance in HCC cells. Furthermore, sorafenib-induced cytotoxic effect in HNF4A-AS1 overexpression cells could be significantly rescued when they were pretreated with the ferroptosis inhibitor ferrostatin-1 (Fer-1), while the autophagy inhibitor 3-methyladenine (3-MA) and the apoptosis inhibitor ZVAD-FMK (ZVAD) only have partial rescue effect (**Figure 4**A, Figure S3A). Additionally, western blot revealed that overexpression of HNF4A-AS1 failed to modulate sorafenib-induced autophagy in resistant cell lines (Figure 4B, Figure S3B). Flow cytometry using AnnexinV/PI also showed that overexpression of HNF4A-AS1 did not increase sorafenib-induced apoptosis in the resistant cell lines (Figure 4C, Figure S3C).

Ferroptosis is a form of cell death mediated by Fenton-reaction-induced lipid peroxidation. The increase in reactive oxygen species (ROS), lipid peroxidation (LPO), and the final production of malondialdehyde (MDA) represent the augmentation of ferroptosis, while glutathione (GSH) is reduced accordingly (6). Our measurement of MDA indicated that the overexpression of HNF4A -AS1 enhanced MDA production following stimulation with sorafenib, and this process was inhibited by the ferroptosis inhibitor Fer-1 (Figure 4D, Figure S3D). While the results of GSH measurement confirmed that overexpression of HNF4A-AS1 significantly reduced GSH levels owing to enhanced ferroptosis, which could be elevated by Fer-1 inhibition of ferroptosis in cells (Figure 4E, Figure S3E). Subsequently, fluorescence microscopy of C11-BODIPY-labeled LPO revealed that overexpression of HNF4A-AS1 increased sorafenib-induced LPO, which was counteracted by Fer-1 (Figure 4F, Figure S3F). Flow cytometry analysis using the DCFH-DA probe also demonstrated that HNF4A-AS1 promoted sorafenib-induced ROS production, and this process was inhibited by Fer-1 (Figure 4G, Figure S3G). Subsequent determination of ferroptosis by MDA, GSH, LPO, and ROS in parental and sorafenib-resistant cells demonstrated that the resistant cell lines were more tolerant to sorafenib-induced ferroptosis (Figure 4H-K, Figure S3H-K). Furthermore, downregulation of HNF4A-AS1 in parental cells using siRNA attenuated sorafenib-induced MDA and improved GSH levels, implying that the reduction of HNF4A-AS1 facilitated tolerance to sorafenib by inhibiting ferroptosis (Figure 4L). Additionally, given that erastin serves as a classical inducer of ferroptosis, we investigated whether HNF4A-AS1 exerted a similar effect on erastin-induced ferroptosis. Results from MDA and GSH measurements revealed that downregulation of HNF4A-AS1 also conferred resistance to erastin-induced ferroptosis in parental cells (Figure 4M).

In conclusion, our findings suggest that HNF4A-AS1 mediates sorafenib resistance, primarily through ferroptosis.

### HNF4A-AS1 depletion inhibits sorafenib-induced ferroptosis via decreasing intracellular PUFA content in HCC cells

**Figure 5:**
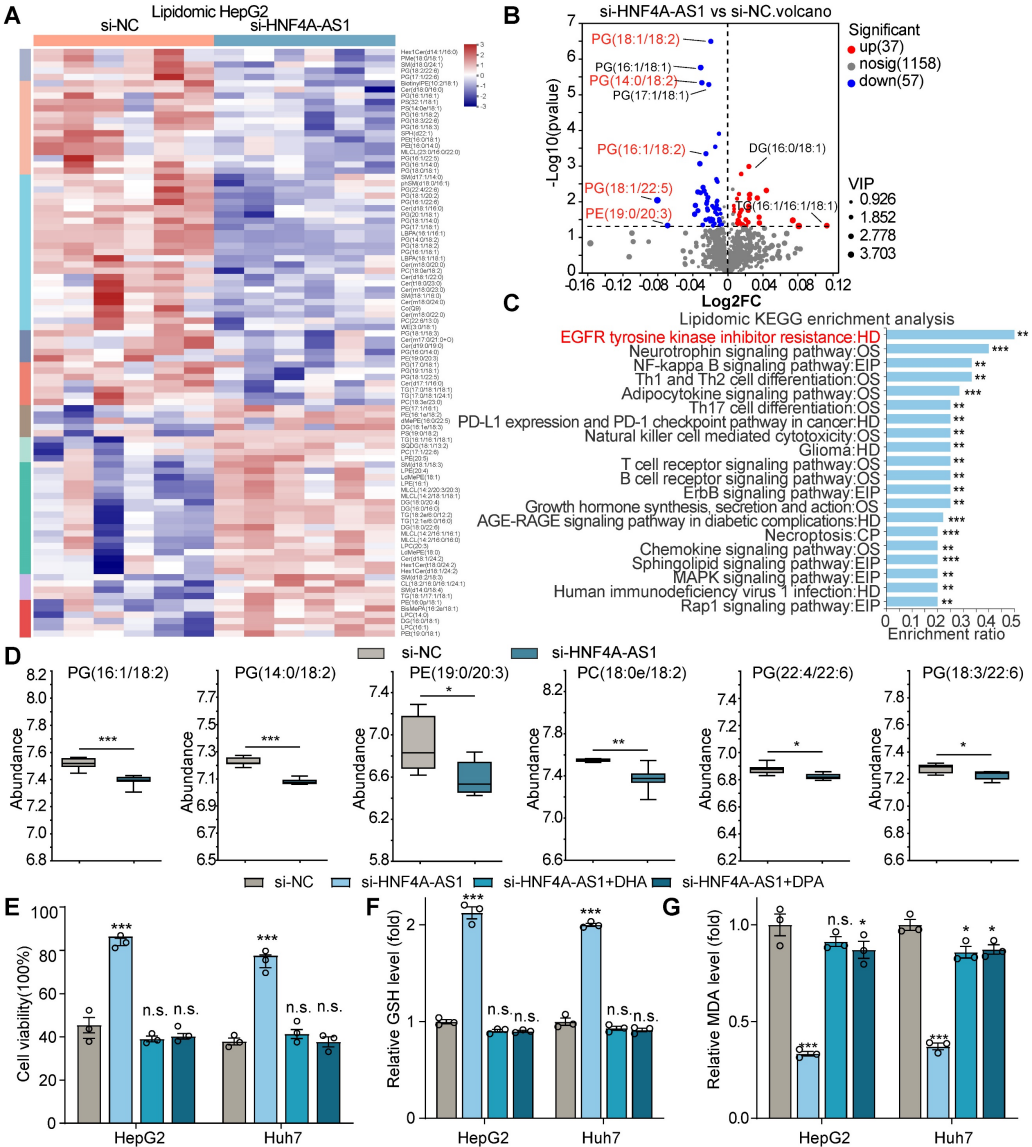
** HNF4A-AS1 regulates PUFA metabolism and affects sorafenib-induced ferroptosis.** (A) Heatmap plot of lipidomic analysis comparing HepG2 cells transfected with si-NC or si-HNF4A-AS1. n = 6. (B) Volcano plot of lipidomic profiling from (A). |FC| > 1, *p*value < 0.05, OPLS-DA VIP > 1. The text shown in red represents lipids containing PUFA. (C) KEGG analysis of lipidomic metabolites from (A). (D) Abundance of different phospholipids that contain PUFA are shown based on (A). (E-G) Cell viability (E), GSH (F), and MDA (G) assays of HepG2 and Huh7 cells transfected with si-NC or si-HNF4A-AS1 and treated with DHA or DPA (50 μM) under sorafenib treatment (10 μM). DHA: docosahexaenoate. DPA: docosapentaenoate. VIP: Variable importance in the projeciton. Data shown are means ± SD from biological triplicates. *:*P* < 0.05, **:*P* < 0.01, ***:*P* < 0.001, n.s.: *P* > 0.05.

Ferroptosis, triggered by iron-dependent lipid peroxidation, is known to be influenced by changes in lipid composition 23. Therefore, we hypothesized that HNF4A-AS1, as a lipid metabolism-related lncRNA, might regulate sorafenib resistance by modulating lipid content. We performed LC-MS lipidomic profiling after HNF4A-AS1 depletion in HepG2 cells (**Figure 5**A). Remarkably, our findings indicated that HNF4A-AS1 downregulation caused a significant reduction in the levels of various phospholipids, which contain polyunsaturated fatty acids (PUFA), such as PG(16:1/18:2), PG(14:0/18:2), PE(19:0/20:3), PC(18:0e/18:2), PG(22:4/22:6), PG(18:3/22:6), PG(18:2/22:6), PG(18:1/22:5), PG(18:1/22:5), PG(18:1/18:2), PG(17:1/22:6), PG(16:1/22:6), PG(16:1/22:5), and PG(16:1/18:3) (Figure 5B-D, Figure S4A). Interestingly, KEGG pathway analysis of the differentially regulated metabolites from lipidomic profiling revealed a strong association with EGFR tyrosine kinase inhibitor resistance (Figure 5C), which is consistent with our previous findings. These results provide valuable insights into the profound effect of HNF4A-AS1 on lipid homeostasis in HCC cells.

For further verify the role of PUFA in regulation of ferroptosis by HNF4A-AS1, we employed Oil Red O staining to assess the increase in intracellular lipids upon supplementation with exogenous PUFA, docosahexaenoate (DHA), and docosapentaenoate (DPA) (Figure S4B). Subsequently, we conducted a series of assays, including cell viability, MDA, GSH, and C11-BODIPY assays, to measure the extent of ferroptosis. The results revealed that the introduction of exogenous PUFAs, DHA and DPA, effectively rescued the diminished ferroptosis caused by si-HNF4A-AS1 in the parental cell lines, as determined by decreased cell viability and GSH levels, and increased MDA and LPO levels (Figure 5E-G, Figure S4C-D).

In addition, we investigated whether HNF4A-AS1 regulate glycolysis, which is generally considered to be a key metabolic factor associated with sorafenib resistance in HCC cells. Our experiments involving knockdown or overexpression of HNF4A-AS1 showed no significantly impact on the expression levels of several glycolysis-related genes (GLUT1, PKM2, ENO1, LDHA), as confirmed by western blot and RT-qPCR analysis (Figure S5A-D). Furthermore, glucose uptake assay and lactate production assay also demonstrated that HNF4A-AS1 did not affect glycolysis in hepG2 cells (Figure S5E-F).

Collectively, these results strongly indicated that HNF4A-AS1 regulates sorafenib resistance by modulating PUFA metabolism.

### HNF4A-AS1 inhibits PUFA catabolism by decreasing DECR1 expression

**Figure 6:**
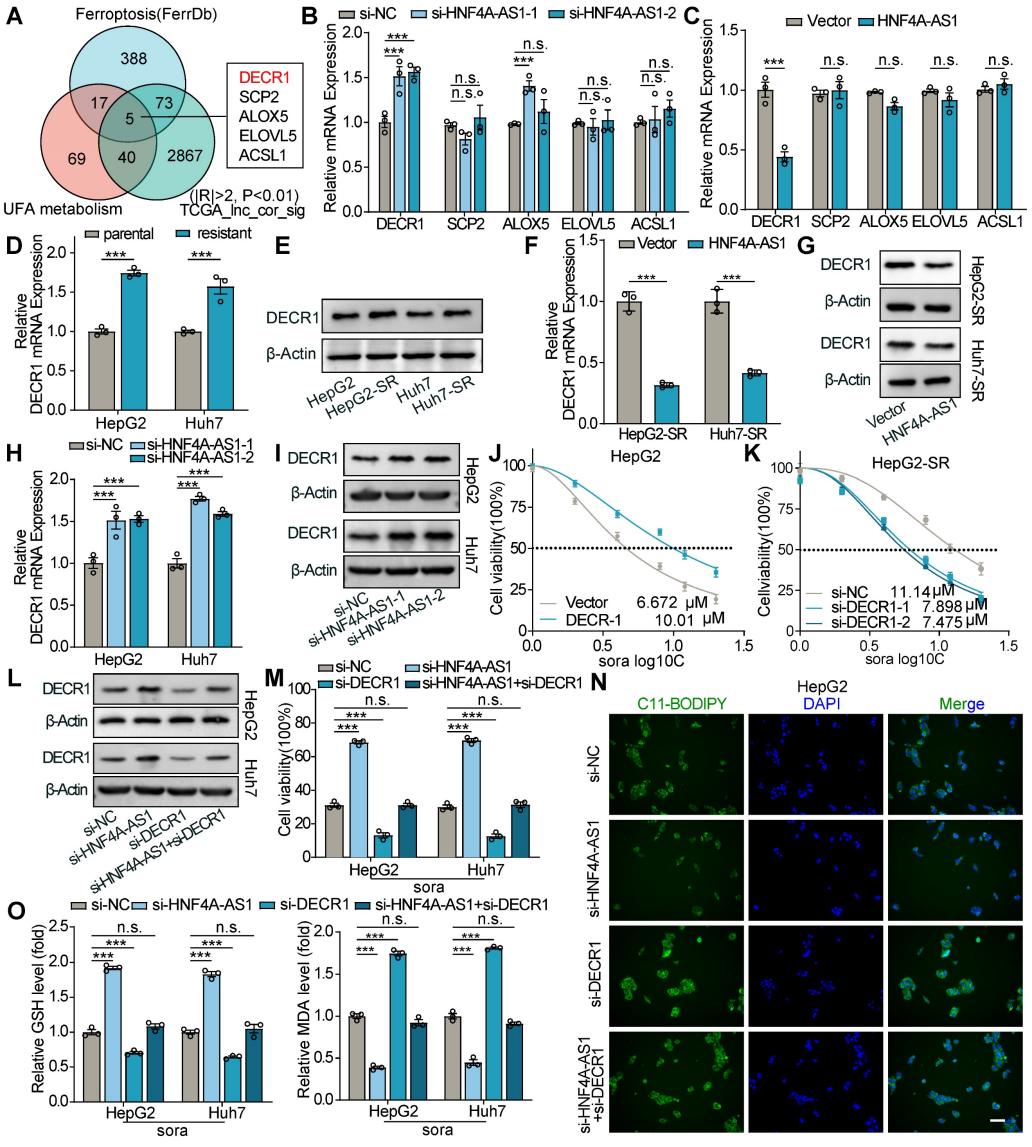
**HNF4A-AS1 mediates sorafenib-induced ferroptosis tolerance through DECR1.** (A) Venn diagram showing the identification of HNF4A-AS1-correlated genes that are involved in unsaturated fatty acid (UFA) metabolism and associated with ferroptosis. (B) The expression of five candidate genes in HepG2 cells transfected with si-NC or si-HNF4A-AS1. (C) Changes in the expression of five candidate genes in HepG2-SR cells overexpressing vector or HNF4A-AS1 plasmid. (D-E) RT-qPCR and western blot analyses of DECR1 expression in parental and sorafenib-resistant HepG2 and Huh7 cells. (F-G) RT-qPCR and western blot analyses of DECR1 expression in HepG2-SR and Huh7-SR cells overexpressing vector or HNF4A-AS1. (H-I) RT-qPCR and western blot analyses of DECR1 expression in HepG2 and Huh7 cells transfected with si-NC or si-HNF4A-AS1. (J-K) IC50 values of sorafenib were measured in HepG2 cells overexpressing HNF4A-AS1 and HepG2-SR cells with si-HNF4A-AS1 interference. (L) Protein levels of DECR1 in HepG2 and Huh7 cells transfected with si-NC, si-HNF4A-AS1, si-DECR1, or co-transfected with both. (M-O) Cell viability (M), LPO (N), GSH, and MDA (O) levels in HepG2 and Huh7 cells transfected with si-NC, si-HNF4A-AS1, si-DECR1, or co-transfected with both under sorafenib treatment (10 μM). Sora: sorafenib. UFA: unsaturated fatty acid. Data shown are means ± SD from biological triplicates. ***:*P* < 0.001, n.s.: *P* > 0.05.

To further elucidate the molecular mechanism by which HNF4A-AS1 leads to a decrease in PUFA, intersection analysis of genes associated with HNF4A-AS1 correlated genes in LIHC, ferroptosis (genesets from FerrDb (http://www.zhounan.org/ferrdb/current)), and UFA metabolism (Supplementary table S1) was performed. Five candidate genes (DECR1, SCP2, ALOX5, ELOVL5, and ACSL2) were identified, and their mRNA levels were measured by RT-qPCR after the downregulation or overexpression of HNF4A-AS1. The results showed that only DECR1 exhibited the strongest correlation with HNF4A-AS1 expression and was negatively regulated by HNF4A-AS1 (**Figure 6**A-C). Interestingly, analysis of the Gepliver database revealed consistent upregulation of DECR1 expression in various liver diseases, including NAFLD, liver cirrhosis, and HCC (Figure S6A).

Next, we examined DECR1 mRNA and protein expression levels in the parental and resistant cells. The results showed that DECR1 expression was higher in sorafenib-resistant cells than in the corresponding parental cells (Figure 6D-E). Additionally, HNF4A-AS1 overexpression decreased both the mRNA and protein expression of DECR1 in resistant cell lines (Figure 6F-G), whereas downregulation of HNF4A-AS1 increased DECR1 expression (Figure 6H-I). These results confirm the negative regulatory relationship between HNF4A-AS1 and DECR1. Subsequently, we investigated the potential association between DECR1 expression and sorafenib resistance in HCC cells. Firstly, we assessed the efficiency of DECR1 knockdown and overexpression using RT-qPCR and western blot (Figure S6B-E). IC50 experiments revealed that overexpression of DECR1 increased the resistance of HCC cells to sorafenib (Figure 6J, Figure S6F), whereas downregulation of DECR1 had the opposite effect (Figure 6K, Figure S6G). Previous studies have reported that elevated DECR1 levels in prostate cancer promote the oxidation of PUFA, leading to a reduction in intracellular PUFA content and the subsequent development of ferroptosis resistance 24,25. Similarly, in our investigation, we observed that overexpression of DECR1 diminished sorafenib-induced ferroptosis in HCC cells, and this process was rescued by supplementing exogenous PUFA (DHA or DPA), as determined by cell viability, LPO, MDA, and GSH (Figure S4H-J). These findings highlight the critical role of DECR1 in modulating the intracellular PUFA composition and its contribution to sorafenib resistance.

Next, we investigated whether DECR1 was an intermediary target involved in the regulation of sorafenib-induced ferroptosis by HNF4A-AS1. At the protein level, we observed that the knockdown of HNF4A-AS1 led to an increase in DECR1 expression. However, this effect was reversed when si-DECR1 was transfected simultaneously (Figure 6L). Subsequently, using cell viability, LPO, MDA, and GSH assays, we discovered that transfection with si-HNF4A-AS1 reduced the sensitivity to sorafenib-induced ferroptosis. Moreover, co-transfection with si-DECR1 and si-HNF4A-AS1 reversed resistance to ferroptosis (Figure 6M-O).

In conclusion, the effect of HNF4A-AS1 on sorafenib-induced ferroptosis was mediated by DECR1.

### HNF4A-AS1 promotes degradation of DECR1 mRNA via METTL3-dependent m6A methylation

Next, we investigated how HNF4A-AS1 regulates the expression of DECR1. Through luciferase assays and polysome profiles, we found no evidence that HNF4A-AS1 regulates DECR1 transcription and translation (Figure S7A-D). However, in the RNA remaining assay, we observed that HNF4A-AS1 overexpression decreased the mRNA stability of DECR1, whereas si-HNF4A-AS1 had the opposite effect (Figure S7E-H). Considering the established role of m6A modifications in RNA degradation, we hypothesized that HNF4A-AS1 modulates DECR1 expression via m6A-mediated mRNA degradation.

**Figure 7:**
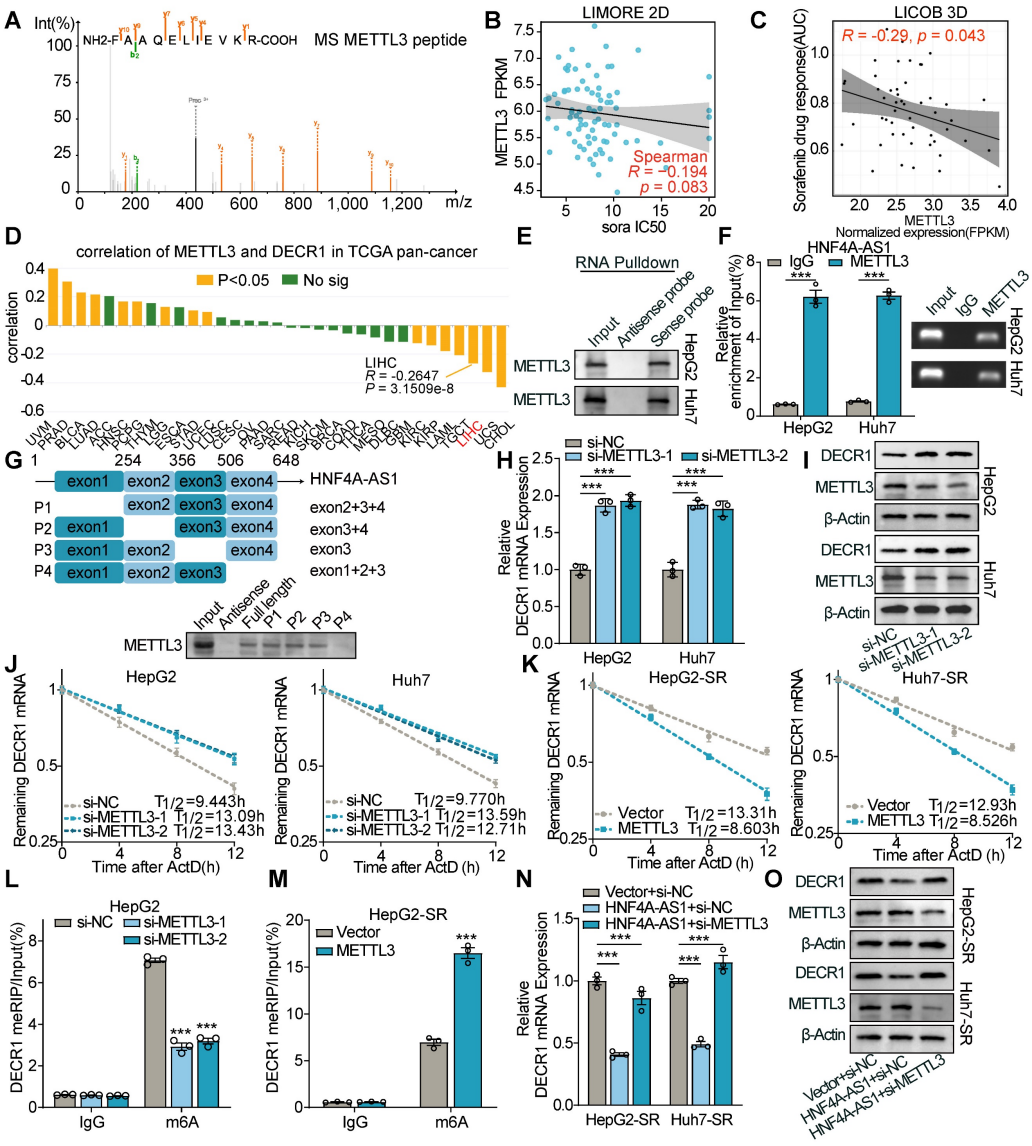
**METTL3-mediated m6A modification decreases DECR1 mRNA stability.** (A) Mass spectrum (MS) peptides of METTL3 following biotin-labeled RNA pulldown using HNF4A-AS1 sense and antisense probes. (B-C) LIMORE and LICOB databases showing the correlation between METTL3 expression and sorafenib response. Higher IC50 and AUC indicate lower sorafenib response. (D) Correlation between METTL3 and DECR1 expression in TCGA pan-cancer datasets, including liver hepatocarcinoma (LIHC). (E) Western blot analysis of METTL3 following biotin-labeled RNA pulldown using HNF4A-AS1 sense and antisense probes. (F) RIP assay performed with anti-METTL3 and anti-IgG antibodies. Detection of HNF4A-AS1 was performed by RT-qPCR in HepG2 and Huh7 cells, followed by visualization on 2% agarose gel. (G) Western blot of METTL3 following biotin-labeled RNA pulldown using full-length or truncated fragments of HNF4A-AS1 in HepG2 cells. (H-I) RT-qPCR and western blot analyses of DECR1 expression in HepG2 and Huh7 cells transfected with si-METTL3. (J) RNA stability assay of DECR1 in HepG2 and Huh7 cells transfected with si-NC or si-METTL3. (K) RNA stability assay of DECR1 in HepG2-SR and Huh7-SR cells transfected with vector or METTL3 plasmids. (L-M) MeRIP-PCR of DECR1 mRNA using anti-m6A or anti-IgG antibodies in HepG2 and HepG2-SR cells transfected with si-NC/si-METTL3 or vector/METTL3, respectively. (N-O) RT-qPCR and western blot analyses of DECR1 in HepG2 and Huh7 cells transfected with vector+si-NC, HNF4A-AS1+si-NC, or HNF4A-AS1+si-METTL3. ActD: actinomycin D. HepG2-SR: sorafenib-resistant hepG2. Huh7-SR: sorafenib-resistant huh7. Data shown are means ± SD from biological triplicates. ***:*P* < 0.001, n.s.: *P* > 0.05.

Therefore, we performed an RNA pulldown assay using biotin-labeled HNF4A-AS1 sense and antisense probes and detected the pulled proteins using mass spectrometry (MS). The results revealed that HNF4A-AS1 binds to METTL3 (**Figure 7**A and Supplementary Table S2). The LIMORE and LICOB databases were used to predict the impact of METTL3 on the response to sorafenib. The results demonstrated that lower METTL3 expression was associated with a poorer response to sorafenib (higher IC50 and AUC) (Figure 7B-C). Additionally, TCGA pan-cancer data revealed a negative correlation between METTL3 and DECR1 expression in LIHC (Figure 7D). Therefore, we hypothesized that it is METTL3 that mediated the regulation of DECR1 by HNF4A-AS1. Next, we validated the interaction between METTL3 and HNF4A-AS1 by performing western blot following RNA pulldown (Figure 7E). In addition, the RIP experiment performed using the METTL3 antibody also verified this binding (Figure 6F). Furthermore, the catRAPID website (http://service.tartaglialab.com/page/catrapid_group) predicted that METTL3 interacts with HNF4A-AS1 (Figure S7I). Deletion-mapping analyses revealed that the 506-648nt region was necessary for the binding of HNF4A-AS1 to METTL3 (Figure 7G).

M6A is a species-conserved RNA post-transcriptional modification that causes RNA stability, splicing, transport, and localization 26. Therefore, we investigated whether DECR1 could be modified by METTL3-mediated m6A modifications. Using the SRAMP algorithm (http://www.cuilab.cn/sramp), we identified two highly confident m6A sites in DECR1 mRNA with an AGACU motif located at positions 481 and 1442 (Figure S7J). Meanwhile, RNA modification database RM2Target (http://m6a2target.canceromics.org/#/) also demonstrated that METTL3 functions as a m6A “writer” of DECR1 mRNA, and negatively regulates DECR1 expression in HEK293T cell, as determined by RNA-seq and meRIP-seq (Figure S7J). Interestingly, western blot and RT-qPCR experiments also validated that si-METTL3 enhanced the expression of DECR1 (Figure 7H-I), while METTL3 overexpression had the opposite effect (Figure S7K-L). Considering that HNF4A-AS1 negatively regulated DECR1 mRNA stability (Figure S7E-F), we hypothesized that METTL3 influences DECR1 expression by affecting the stability of its mRNA. Interestingly, we investigated the RNA remaining assay and demonstrated that knockdown of METTL3 enhanced the mRNA stability of DECR1 in the presence of the RNA polymerase inhibitor ActD (Figure 7J), whereas overexpression of METTL3 decreased its stability (Figure 7K). Moreover, the MeRIP assay revealed that m6A modification of DECR1 was decreased following METTL3 interference (Figure 7L), while the reverse effect was observed with METTL3 overexpression (Figure 7M).

To verify the HNF4A-AS1/METTL3/DECR1 axis, we performed a rescue assay. The results of western and RT-qPCR showed that si-METTL3 restored the decreased expression of DECR1 under HNF4A-AS1 overexpression (Figure 7N-O). Additionally, the overexpression of METTL3 reversed the increase in DECR1 expression caused by si-HNF4A-AS1 (Figure S7M-N).

As previous studies have reported that lncRNAs are subject to regulation by m6A modification 27,28, we examined whether METTL3 affects the expression of HNF4A-AS1. The results showed that METTL3 knockdown or overexpression did not induce changes in HNF4A-AS1 expression (Figure S7O-P).

Collectively, these findings indicated that METTL3-dependent m6A modification mediates the regulatory effect of HNF4A-AS1 on DECR1 expression.

### YTHDF3 is required for the HNF4A-AS1-mediated mRNA degradation of DECR1

**Figure 8:**
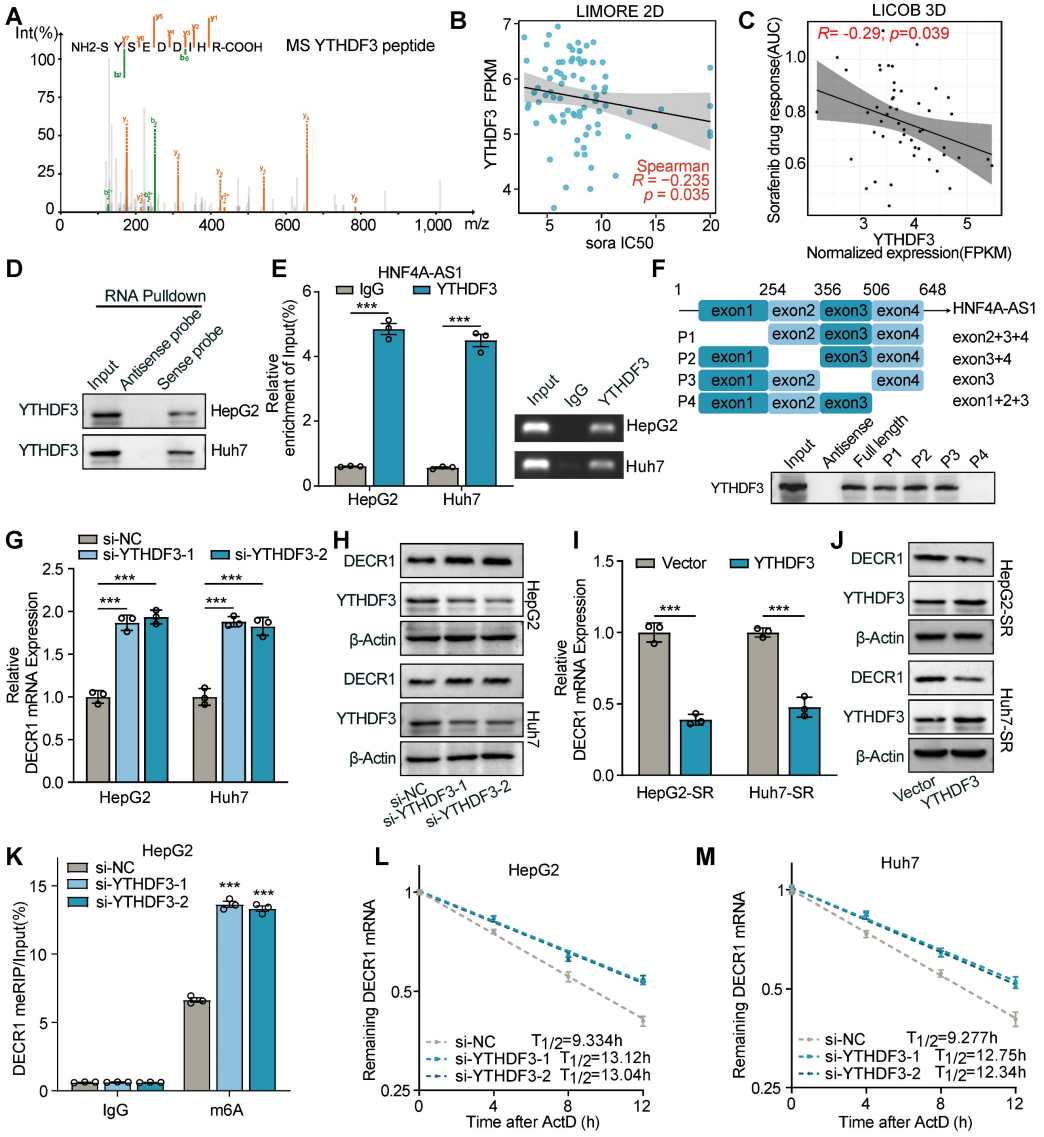
**YTHDF3 decreases DECR1 mRNA stability via m6A modification.** (A) Mass spectrum peptides analysis of YTHDF3 followed by biotin-labeled RNA pulldown using HNF4A-AS1 sense and antisense probes. (B-C) LIMORE and LICOB databases showing the correlation between YTHDF3 expression and sorafenib response. Higher IC50 and AUC indicate lower sorafenib response. (D)Western blot analysis of YTHDF3 following biotin-labeled RNA pulldown using HNF4A-AS1 sense and antisense probes. (E) RIP assay performed with anti-YTHDF3 and anti-IgG antibodies in HepG2 and Huh7 cells. (F) Western blot analysis of YTHDF3 following biotin-labeled RNA pulldown using full-length or truncated fragments of HNF4A-AS1 in HepG2 cells. (G-H) RT-qPCR and western blot analyses of DECR1 expression in HepG2 and Huh7 cells transfected with si-YTHDF3. (I-J) RT-qPCR and western blot analyses of DECR1 expression in HepG2-SR and Huh7-SR cells transfected with vector or YTHDF3 plasmid. (K) meRIP-PCR of DECR1 mRNA using anti-m6A or anti-IgG antibodies in HepG2 cells transfected with si-NC or si-YTHDF3. (L-M) RNA stability assay of DECR1 in HepG2 and Huh7 cells transfected with si-NC or si-YTHDF3 by RT-qPCR. ActD: actinomycin D. HepG2-SR: sorafenib-resistant hepG2. Huh7-SR: sorafenib-resistant huh7. Data shown are means ± SD from biological triplicates. ***:*P* < 0.001, n.s.: *P* > 0.05.

Interestingly, in our pulldown-MS results of HNF4A-AS1 binding proteins, we not only identified METTL3 but also discovered the presence of YTHDF3 (**Figure 8**A). The predicted binding of YTHDF3 to HNF4A-AS1 was confirmed using the CatRAPID website (Figure S8A). The LIMORE and LICOB databases were then used to predict the impact of YTHDF3 on the response to sorafenib. The results demonstrated that lower YTHDF3 expression was associated with a poorer response to sorafenib in liver cancer (higher IC50 and AUC) (Figure 8B-C). Therefore, we hypothesized that it is YTHDF3 that mediated the regulation of DECR1 by HNF4A-AS1. Subsequently, we performed RNA pulldown-western blot to validate the interaction between HNF4A-AS1 and YTHDF3 (Figure 8D). Additionally, RIP experiments using the YTHDF3 antibody further confirmed the binding of YTHDF3 to HNF4A-AS1 (Figure 8E). Deletion-mapping analyses revealed that the 506-648nt region was necessary for the binding of HNF4A-AS1 to YTHDF3 (Figure 8F).

Subsequently, we investigated the regulatory role of YTHDF3 on the expression of DECR1. We evaluated the interference efficiency of si-YTHDF3 and the overexpression efficiency of the YTHDF3 plasmid using RT-qPCR (Figure S8B-C). RT-qPCR and western blot results demonstrated that knockdown of YTHDF3 led to a increase in DECR1 expression (Figure 8G-H), while overexpression of YTHDF3 decreased its expression (Figure 8I-J). Additionally, our meRIP-PCR results revealed that YTHDF3 interference increased m6A modification of DECR1 mRNA (Figure 8K), while its overexpression had the opposite effect (Figure S8D). YTHDF3 has been reported to modulate m6A-mediated mRNA stability 29,30. Consistently, our results showed that downregulation of YTHDF3 led to an increase in the mRNA stability of DECR1 (Figure 8L-M), while overexpression of YTHDF3 had the opposite effect (Figure S8E-F). Furthermore, rescue experiments were conducted to explore the HNF4A-AS1/YTHDF3/DECR1 axis. Our results revealed that overexpression of HNF4A-AS1 reduced the expression of DECR1, whereas knockdown of YTHDF3 suppressed this effect (Figure S8G-H). Similarly, knockdown of HNF4A-AS1 increased the expression of DECR1, which was rescued by the overexpression of YTHDF3 (Figure S8I-J). Finally, we determined that the alteration in YTHDF3 expression did not affect the expression level of HNF4A-AS1, despite their interaction (Figure S8K-L).

In summary, our findings highlight the role of YTHDF3 as a modulator of the m6A-mediated degradation of DECR1 mRNA by HNF4A-AS1.

### HNF4A-AS1 and PUFA synergistically enhanced the effect of sorafenib in HCC

**Figure 9:**
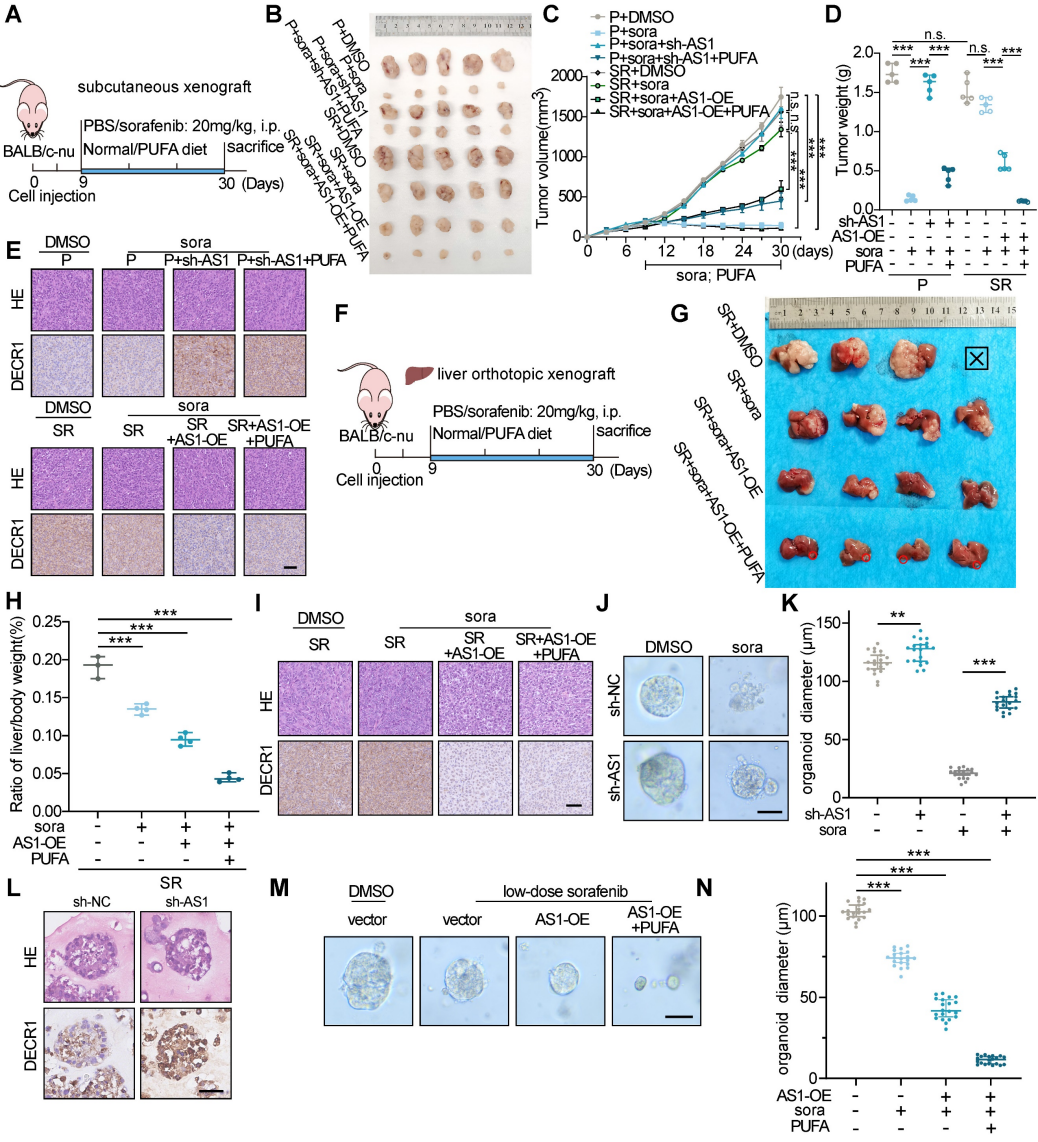
** The impact of HNF4A-AS1 and PUFA on sorafenib sensitivity was assessed in xenografts and organoids.** (A) Schematic diagram of the subcutaneous xenograft model. (B-D) Representative images (B), growth curve (C), and tumor weight (D) of xenografts in the eight treatment groups over a period of 30 days are shown. (E) HE and IHC staining of the subcutaneous xenografts in (A). Scale bar:50 μm. (F-I) Schematic diagrams (F), representative images (G), ratio of liver/body weight (H), HE staining, and IHC staining (I) of the liver orthotopic xenografts. Scale bar:50 μm. (J-L) Representative images (J), diameter measurement (K), HE and IHC staining (L) of PDO transfected with LV-sh-HNF4A-AS1 with or without sorafenib (10 μM). Scale bar:50 μm. (M-N) Representative images and diameter measurement of the PDO with low-dose sorafenib treatment (2 μM). Scale bar:50 μm. P: parental hepG2 cells, SR: sorafenib-resistant hepG2 cells. Sora: sorafenib. Data shown are means ± SD from biological triplicates. *:*P* < 0.05, **:*P* < 0.01, ***:*P* < 0.001, n.s.: *P* > 0.05.

To further investigate the impact of HNF4A-AS1 on the sorafenib response in HCC *in vivo*, we performed subcutaneous xenografts with HCC cells stably transfected with Lv-lnc-HNF4A-AS1 or Lv-sh-HNF4A-AS1 in nude mice (**Figure 9**A). Consistent with our previous *in vitro* findings, HepG2-SR-derived xenograft tumors exhibited greater tolerance to sorafenib treatment than parental xenograft tumors, as indicated by tumor volume and weight. Additionally, downregulation of HNF4A-AS1 weakened sorafenib response in parental cell-derived xenograft tumors, while PUFA supplementation rescued this effect. On the other hand, overexpression of HNF4A-AS1 reduced sorafenib resistance in resistant cell-derived xenograft tumors, and PUFA supplementation synergistically enhanced the cytotoxic effect of sorafenib (Figure 9B-D). Immunohistochemistry (IHC) analysis confirmed the negative regulation of DECR1 by HNF4A-AS1 *in vivo* (Figure 9E). The liver orthotopic xenograft tumors displayed a similar trend in the liver/body weight ratio and IHC staining (Figure 9F-I). HNF4A-AS1 overexpression significantly inhibited the resistance to sorafenib and PUFA supplementation synergistically potentiated this effect, as evidenced by a reduction in tumor volume and a decrease in the liver/body weight ratio (Figure 9G-H). Furthermore, IHC assay demonstrated that elevated HNF4A-AS1 levels were associated with diminished DECR1 expression in liver orthotopic xenograft tumors (Figure 9I). Subsequently, we examined the effect of HNF4A-AS1 on the efficacy of sorafenib in HCC patient-derived organoids (PDO). The results demonstrated that downregulation of HNF4A-AS1 reduced the sensitivity of PDO cells to sorafenib, as indicated by changes in organoid diameter (Figure 9J-K). Furthermore, IHC staining revealed that sh-HNF4A-AS1 increased DECR1 expression in PDO (Figure 9L). Lastly, we treated PDO with low-dose sorafenib and found that overexpression of HNF4A-AS1 increased the sensitivity of PDO to sorafenib, with further enhancement observed upon the addition of PUFA (Figure 9M-N).

In conclusion, our findings indicate that HNF4A-AS1 overexpression facilitates the efficacy of sorafenib in HCC and that its combination with PUFA supplementation has a synergistic effect.

## Discussion

Despite recent studies identifying the significant roles of lncRNAs in sorafenib resistance in HCC, the unique function of lipid metabolism-related lncRNAs, if any, remains unclear. In this study, we discovered a lipid metabolism-related and liver-specific lncRNA, HNF4A-AS1, which was downregulated in HCC and sorafenib-resistant cells. Notably, depletion of HNF4A-AS1 decreased the accumulation of intracellular PUFA, thereby promoting resistance to sorafenib-induced ferroptosis. Furthermore, we found that HNF4A-AS1 downregulation modulated PUFA metabolism by enhancing DECR1 expression by decreasing METTL3/m6A/YTHDF3 mediated mRNA degradation.

The tissue-specific and cell-specific expression patterns of lncRNAs emphasize their potential as biomarkers and for targeted clinical applications with minimal adverse effects 14,16. Swhtr, a heart-specific lncRNA, is not necessary for normal heart development and function but is essential for compensatory cardiac response following myocardial infarction 31. The deficiency of HOXA11os, a colonic myeloid cells-specific lncRNA, leads to the generation of mitochondrial reactive oxygen species (mtROS) and therefore the development of spontaneous intestinal inflammation and increased susceptibility to colitis 32. Researchers have attempted to employ machine learning on single-cell RNA-seq data for the identification of a detailed subtype- and cell-type-specific expression of lncRNAs in breast cancer 33. In our study, through bioinformatics analyses, we discovered that HNF4A-AS1, a lipid metabolism-related and relatively liver-specific lncRNA, is downregulated in HCC and contributes to sorafenib resistance. Consistent with previous studies 34, we revealed that downregulation of HNF4A-AS1 is associated with advanced clinical stages and poorer prognosis in patients with HCC. However, we provided a more profound exploration into the role of HNF4A-AS1 in sorafenib resistance and its potential therapeutic value.

The insensitivity of different programmed cell death plays a crucial role in conferring resistance to sorafenib in HCC, especially autophagy, apoptosis, and ferroptosis 35. For example, SCAP contributes to sorafenib resistance by inhibiting AMPK-mediated autophagy 7. Iron deficiency mediates the upregulation of HIF-1α-regulated apoptotic proteins and hampers apoptosis, consequently fostering resistance to sorafenib 36. Loss of the leukemia inhibitory factor receptor (LIFR) confers HCC proliferation and sorafenib-induced ferroptosis resistance by increasing the expression of the iron-sequestering cytokine LCN2 37. URB1-AS1 is overexpressed in sorafenib-resistant HCC samples and dampens sorafenib-induced ferroptosis by triggering ferritin phase separation 38. While previous studies have implicated lncRNAs in sorafenib resistance 12,38, our findings uniquely link lipid metabolism-related lncRNAs to the modulation of ferroptosis, offering a novel perspective on HCC treatment resistance. We demonstrated that depletion of HNF4A-AS1 modulates PUFA accumulation, leading to a reduction in ferroptosis and the subsequent development of sorafenib resistance.

Lipid metabolism is closely linked to ferroptosis, which is primarily characterized by excessive peroxidation of PUFAs 39,40. In gastric cancer, decreased expression of ELOVL5 and FADS1, enzymes involved in PUFA biosynthesis, leads to resistance to ferroptosis, which is reversed by the addition of exogenous PUFA *in vitro* 9. Additionally, a PUFA-rich diet has been observed to promote ferroptosis and significantly suppress tumor growth compared with a MUFA-rich diet in mice 39. As identified lipid-metabolism related lncRNA, our results from *in vitro* and *in vivo* experiments suggested that a potential treatment regimen for patients with HCC could involve combining sorafenib treatment with HNF4A-AS1 overexpression and a PUFA-rich diet.

Recent studies have highlighted the crucial role of m6A in regulating various biological processes, including cancer progression and drug resistance 26,41. Specifically, alterations in m6A modification have been implicated in sorafenib resistance in HCC, indicating a complex interplay between epigenetic modifications and therapeutic outcomes 42-44. Down-regulation of METTL3, a primary m6A methyltransferase, has been shown to promotes sorafenib resistance in HCC by reducing FOXO3 mRNA stability through m6A modification, leading to the activation of autophagy-associated pathways and enhanced expression of angiogenesis genes 45. Moreover, the m6A reader YTHDF1 has been identified as a driver of HCC stemness and drug resistance by enhancing m6A-mediated NOTCH1 mRNA stability and translation 46. Nevertheless, the involvement of the m6A-mediated lipid metabolism reprogramming in drug resistance in HCC is still not fully elucidated. In our study, we investigated the role of HNF4A-AS1 in regulating m6A modification of DECR1 mRNA, which ultimately results in mRNA degradation. Specifically, we found that HNF4A-AS1 facilitates m6A modification of DECR1 mRNA by interacting with the m6A "writer" METTL3 and the m6A "reader" YTHDF3, respectively, thereby manipulating intracellular PUFA content.

In conclusion, this study elucidates the loss of lipid metabolism-related and relatively liver-specific HNF4A-AS1 confers sorafenib resistance by reducing m6A-mediated DECR1 degradation, which further contributes to a decrease in intracellular PUFA and therefore inhibits PUFA-dependent ferroptosis. It's suggested that HNF4A-AS1 is a prospective therapeutic target for enhancing the effectiveness of sorafenib.

## Conclusion

In summary, these results revealed a lipid metabolism-related and relatively liver-specific lncRNA HNF4A-AS1 inhibits sorafenib resistance in HCC, by promoting METTL3/m6A/YTHDF3-mediated DECR1 mRNA degradation, leading to intracellular PUFA accumulation, and subsequently, sorafenib-induced ferroptosis. It was suggested that the HNF4A-AS1 was a potentially valuable therapeutic target to enhance the sorafenib treatment response in HCC.

## Methods

### Patients tissue specimens

Twenty pairs of HCC (T) and normal peritumoral (N) tissue specimens were collected from patients without systemic therapy or radiotherapy before surgery in the Department of Hepatobiliary Surgery of Union Hospital, Wuhan, China. Tissues were obtained by surgical resection of the patients. All specimens were independently confirmed by at least two experienced histopathologists according to the criteria of the sixth edition of the TNM classification of the International Union Against Cancer. Part of the excised tissue specimens were immediately frozen in liquid nitrogen after surgical resection, and the residual tissue was fixed in 10% buffered formalin solution and then embedded in paraffin. All samples were collected with written informed consent from the patients, and the study was approved by the local Research Ethics Committee at the Academic Medical Centre of Huazhong University of Science and Technology.

### Xenograft

For the xenograft assay, male BALB/c nude mice aged 4-6 weeks were selected to establish the model. Both the lentivirus-based short hairpin (sh) RNA vector GV493 and the lentivirus-based overexpression RNA vector GV721 was purchased in Shanghai GeneChem Co., Ltd. The interference sequences for HNF4A-AS1 were 5'-GGAGCUGGGAUCUGACACU-3'. According to the manufacturer's instruction, the Lv-lnc-HNF4A-AS1 or Lv-sh-HNF4A-AS1 lentivirus was then diluted with serum-free DMEM medium and used to infect HepG2 cells at a multiplicity of infection of 10. The medium was refreshed after 8 hours. Subsequently, at 72 h post-infection, puromycin (2 μg/ml) was employed to screen out the stable knockdown or overexpression cell lines for 48 h, and then 1 μg/ml puromycin was used for maintenance. Finally, the stably transfected HepG2 cells were harvested (2×10^6^ cells/mouse), washed twice with cold PBS and suspended in a 1:1 mixture of Matrigel (R&D Systems, USA, BME001-10). For subcutaneous HCC mice xenograft model, the cell suspension was injected into the right flank of the mice (n = 5 per group). On the ninth day after cell injection, the mice were intraperitoneally injected with either sorafenib (20 mg/kg, MCE, USA, HY-10201) or a saline solution while being fed a normal diet or a PUFA-rich diet (Jiangsu-Xietong, China). The size of the tumor was measured every three days with a vernier caliper, and the mice were humanely sacrificed under general anesthesia to measure the liver weight and body weight on the 30th day. For orthotopic HCC mice xenograft model, the cell suspension was injected intrahepatically into mice (n = 4 per group). Thirty days after the injection, all mice were humanely sacrificed, and livers were extracted for examination. The observational endpoints considered were clinical endpoints such as significant weight loss and reduced mobility, in addition to the standard methodologies of monitoring. All animal experiments were performed in accordance with the guidelines of the Animal Research Committee of the Academic Medical Center at the Huazhong University of Science and Technology. Animal care and handling strictly adhered to the guidelines set forth by the Institutional Animal Care and Use Committees.

### PDO

The HCC organoid model was based on the previously published protocol 47. In brief, fresh surgical specimens were retrieved from 4℃ tissue preservation solution, washed twice with 1% P/S PBS, and then cut into 1-2 mm^3^. The fragments were dissociated with digestion solution at 37℃ for 30 min, followed by termination of digestion using ice-cold base medium (Advanced DMEM/F12 (Gibco, USA, 12634028), 1% HEPES (Gibco, USA, 15630106), 1% GlutaMAX (Gibco, USA, 35050061), 1% penicillin/streptomycin (Gibco, USA, 15140122)). The digested mixture was filtered through a 100 μm mesh, and then centrifuged at 4℃, 300 g for 5 min. After washing twice with ice-cold base medium, the cells were resuspended in matrigel (R&D Systems, USA, BME001-10) and seeded in a 24-well plate. After solidification of the matrigel, 500μl of initiation medium was added, which was then replaced with expansion medium (EM) after 5 days for continued cultivation. The initiation medium (IM) consists of base medium, 1:50 N21-MAX Media Supplement (50X) (R&D systems, USA, AR008), 1:100 N-2 MAX Media Supplement (100X) (R&D systems, USA, AR009), 1.25mM N-acetyl-L-cysteine (MCE, USA, HY-B0215), 10mM nicotinamide (MCE, USA, HY-B0150), 10nM (Leu15) gastrin I (R&D systems, USA, 3006/1), 50 ng/ml recombinant human EGF (R&D systems, USA, 236-EG), 100 ng/ml recombinant human FGF10 (R&D systems, USA, 345-FG), 25 ng/ml recombinant human HGF (R&D systems, USA, 294-HGN-025/CF), 10 μM forskolin (Tocris, USA, 1099/10), 5 μM A8301 (Tocris, USA, 2939/50), 3nM dexamethasone (Sigma, USA, D4902-25MG) and 10μM Y27632 (MCE, USA, HY-10071). The expansion medium (EM) contains IM without dexamethasone and supplement with 500ng/ml recombinant human r-spondin1 (R&D systems, USA, 4645-RS-100/CF). The organoids were transfected with Lv-lnc-HNF4A-AS1 or Lv-sh-HNF4A-AS1 lentivirus along with their corresponding control lentiviruses. The infection of lentivirus was based on the previous published protocol 48. In brief, organoid single-cell suspension was prepared as by TrypLE. 5 μl lentivirus suspension was applied to 2 × 10^5^ organoid single cells suspended in 500 μl EM with 10 μM Y27632 (without Matrigel) in one well of 24-well plate and incubated at 37°C for 8h. Subsequently, cells were harvested by TrypLE and washed twice with cold Advanced DMEM/F12 before seeding in Matrigel in a 24-well plate. Cells were grown in EM with Y27632 (10 μM) for 72 h.

### Cell culture

HCC cell lines (HepG2, MHCC-97H, SNU-449, Huh7) and a normal liver cell line (MIHA) were purchased from the American Type Culture Collection (ATCC, USA). Cells were cultured in DMEM (Gibco, USA, 11965092) supplemented with 10% FBS (Gibco, USA, 10099158) and 1% penicillin/streptomycin (Gibco, USA, 15140122) at 37 ℃ under 5% CO2. All cell lines were confirmed three months before the beginning of the study based on a short tandem repeat method and tested negative for mycoplasma contamination. The fatty acids, docosahexaenoate (DHA, MCE, HY-B2167) and docosapentaenoate (DPA, MCE, HY-115437), were prepared using BSA-containing PBS to obtain a fatty acid/BSA ratio of 4:1 (w:w). Aliquoted fatty acids were safely stored at -20℃, shielded from light exposure, and used immediately after thawing. For PUFA treatment assay, cell lines were exposed to 50 μM of fatty acids conjugated with BSA for a duration of 24 hours. Subsequently, the cells were subjected to further analysis.

### Resistance cell lines development

HepG2 and Huh7 resistant cell lines, HepG2-SR and Huh7-SR, were established through a stepwise process using sorafenib (MCE, USA, HY-10201). Resistance was induced by gradually increasing the concentration of sorafenib in complete culture medium. The process started with a concentration of 0.1 μM and after each passage, the concentration of sorafenib was incrementally increased. The concentrations used in the increments were 0.5, 1.0, 2.0, 4.0, 8.0 μM. Finally, the cells were stabilized at a concentration of 12 μM sorafenib. Throughout the process, the cells were maintained in a stable state to establish and characterize the resistant cell lines, HepG2-SR and Huh7-SR.

### Transfection

For siRNA transfection, cells (4 × 10^5^) were seeded in a six-well plate and allowed to adhere prior to transfection. For each well, 200 μl Opti-MEM™ I Reduced Serum Medium (Gibco, USA, 51985091) was used to dilute specific siRNAs and was then mixed with 10 μl INTERFERin (Polyplus, French, 101000028). After vortexing for 5 seconds, the mixtures were incubated at room temperature for 15 minutes, and subsequently being slowly added to the medium. The final concentration of siRNA was 10 nM. After a 24-hour incubation period, the culture medium was replaced with fresh medium. The siRNA sequences (Genepharma, China) are listed in Supplementary Table S3.

### Lipidomics

Lipidomic analysis was carried out by Majorbio (Shanghai, China). Cells (1×10^7^) were subjected to lipid extraction, for which 80 µL of methanol and 400 µL of MTBE were used. The process involved grinding (6 min, -10 ℃ and 50 Hz), ultrasonication (30 minutes, 5 ℃, 40 kHz), and centrifugation (15 min, 4 ℃, 13,000 g). Next, 350 µL of the upper phase lipid extracts was transferred to EP vials and dried in a vacuum concentrator. Prior to another round of ultrasonication (5 min, in ice water, 40 kHz), the lipid extracts were re-dissolved in 100 µL of an isopropanol: acetonitrile (1:1, v/v) solution. Then, 80 µL of supernatant was gently transferred to sample vials for LC-MS/MS analysis.

For the UHPLC-MS/MS analysis, a Thermo UHPLC-Q Exactive HF-X Vanquish Horizon system equipped with an Accucore C30 column was used to perform lipid chromatographic separation. The mobile phases included 10 mM ammonium acetate in ACN:H2O (1:1, v/v) with 0.1% (v/v) formic acid (solvent A) and 2 mM ammonium acetate in ACN:IPA: H2O (10:88:2, v/v/v) along with 0.02% (v/v) formic acid. Standard injection parameters were as follows: volume: 2 µL, flow rate: 0.4 ml/min, and column temperature: 40 ℃. In both positive and negative ion modes, data were acquired using a Thermo Q-Exactive Mass Spectrometer fitted with an electrospray ionization (ESI) source. Data acquisition was executed in data-dependent acquisition (DDA) mode across a mass range of 200-2000 m/z.

For statistical analysis, the ropls R package (version 1.6.2), hosted on the Majorbio Cloud Platform (https://cloud.majorbio.com), was used for multivariate statistical analysis. An unsupervised method was applied for principal component analysis (PCA) to offer an overview of the lipidomic data and visualize patterns, trends, or outliers. The selection of statistically significant metabolites was based on the OPLS-DA model and Student's *t* test, with a VIP value > 1 and a p-value < 0.05. Phospholipids were identified as important differential metabolites, and selected metabolites were chosen for further study. The differential metabolites were clustered using a complete hierarchical clustering heatmap based on the VIP value from the OPLS-DA.

### Colony formation assay

The cells were seeded in triplicate in 6-well plates at a density of 3000 cells per well. The cells were then treated with the described concentrations of sorafenib. After a period of two weeks, the plates were fixed using 4% polyoxymethylene for 20 min and then stained with 0.1% crystal violet for 20 min.

### Cell cytotoxicity and cell viability Assay

Cell Counting Kit-8 (Hycezmbio, China, HYCCK8) was used to evaluate the inhibitory effects of the drugs on HCC cells. In brief, the cells were seeded in 96-well culture plates at a density of 5000 cells per well in 200 μl of culture medium with certain drugs at different concentration gradients for 72 h at 37°C. CCK-8 solution (10 μl/well) was added and incubated for another 1 h. Then, the optical density (OD) was measured at 450 nm with a microplate reader (Thermofish, USA).

### Glucose uptake assay

Glucose uptake was assessed using the fluorescence-based 2-NBDG assay kit (APExBIO, USA, K2212). Following transfection, the media was replaced with Krebs Ringer Bicarbonate (KRB) buffer containing 2-NBDG (100 µM) and incubated for 30 minutes at 37°C. The cells were then washed with KRB buffer, and fluorescence intensity was measured using flow cytometry.

### Lactate Production Assay

Lactate production was measured using a lactic acid assay kit (NanjingJiancheng, China, A019-2-2). To quantify lactate levels, a standard curve was prepared using known lactate concentrations. After the incubation period, the culture media was collected, and lactate concentration was assessed by measuring absorbance at 530 nm using an ELISA plate reader (Thermo Fisher Scientific, USA). Lactate production was normalized to cell number and determined from the standard curve.

### RT-qPCR

Total RNA was extracted from cells and tissues using RNAiso Plus TRIzol reagent (Takara, Japan, 9109). Reverse transcription of total RNA was performed using PrimeScript® RT Master Mix Perfect Real-Time (Takara, Japan, RR036A). Quantitative real-time PCR was performed with SYBR Premix Ex Taq II (Takara, Japan, RR820A) using the StepOnePlus real-time PCR system (Applied Biosystems, USA). β-actin was used as an internal control, and quantitative analysis of the relative expression levels was performed using the 2^-△△Ct^ method. All the primers (Sangon, China) used are listed in Supplementary Table S4.

### Western blot

Cellular total proteins were extracted using RIPA Lysis Buffer (Beyotime, China, P0013B) according to the manufacturer's instructions. The total protein concentration was measured using the Enhanced BCA Protein Assay Kit (Beyotime, China, P0010S). Western blot was performed using specific antibodies against METTL3, DECR1, GAPDH, β-actin and etc. Information on the antibodies used is shown in Supplementary Table S5.

### Chromatin immunoprecipitation (ChIP)

Chromatin immunoprecipitation was performed using the EZ-ChIP™ ChIP reagent (Millipore, MA, USA) according to the manufacturer's instructions. Cells were seeded in 15 cm plates and subjected to cross-linking with 1% formaldehyde for 20 minutes. After cross-linking, the cells were lysed by Western&IP lysis buffers (Beyotime, China, P0013), and the chromatin was sheared to achieve DNA fragments of 300-500 base pairs using ultrasonic sonication. The resulting lysates were then incubated with HIF-1α antibodies (Proteintech, China, 20960-1-AP) or Rabbit IgG (Proteintech, China, 30000-0-AP) overnight at 4°C to form DNA-protein complexes. The precipitated DNA fragments were analyzed by PCR.

### Polysome profiling assay

Cells were treated with 100 mg/mL of cycloheximide (MCE, USA, HY-12320) at 37℃ for 30 min, followed by pelleting and lysed on ice using polysome lysis buffer. Subsequently, the lysate was obtained, applied onto a 10%-50% (w/v) sucrose gradient solution prepared in lysis buffer, and subjected to centrifugation at 4℃ for a duration of 4 h at 30,000 rpm using an ultracentrifuge Optima L-70 with SW 41 Ti Rotor (Beckman Coulter). The resulting sample was fractionated and divided into 12 fractions, which were then assessed using the Gradient Master software (BioComp Instruments) in conjunction with an EM1-Econo UV monitor (Bio-Rad) and fraction collector FC203B (Gilson). After purification from each fraction using RNAiso Plus TRIzol reagent (Takara, Japan, 9109), the RNA was subjected to RT-qPCR analysis.

### Luciferase assay

Briefly, the sequence of the DECR1 promoter (2kb sequence upstream of the transcription initiation site) was constructed into pGL3-based vectors and then transfected into cell lines. Luciferase activity was measured using a luciferase assay kit (Promega, USA, E1910). Firefly luciferase activity was normalized to the Renilla luciferase activity.

### RNA fluorescence *in situ* hybridization

RNA Fluorescence *in situ* hybridization was performed according to the manufacturer's instructions for the Fluorescent *in Situ* Hybridization Kit (Genepharma, China, C10910). In brief, after blocked in prehybridization buffer for 30 minutes, fixed cells were incubated overnight at 37°C in hybridization buffer containing 2.5 μM biotin-labeled FISH probe (RiboBio, China) and 1 μM Cy3-conjugated streptavidin (Genepharma, China). The next day, cells were incubated with counterstained with DAPI for 10 minutes. Images were captured using a Nikon A1 Laser Scanning Confocal Microscope (Nikon, Japan).

### RNA pull-down assays

Specific steps were carried out in accordance with the instructions in the Pierce™ Magnetic RNA-Protein Pull-Down Kit (Thermo Fisher, USA, 20164). Biotin-labeled probes of HNF4A-AS1 sense and antisense strands were designed and synthesized by Genepharma, China. Cell lysates were prepared using a Western&IP lysis buffers (Beyotime, China, P0013) containing protease and RNase inhibitors. In brief, 50 pmol Biotin-labeled probes bind to 50 μl streptavidin magnetic beads, and incubate with 100 μg cell lysate overnight at 4 °C with rotation. Following the wash and elution process, the binding complexes were separated for use in Western Blot and MS assays.

### RNA stability assay

Cells were cultured in 6-well plates overnight and treated with actinomycin D (MCE, USA, HY-17559) at a final concentration of 5 μg/mL to inhibit gene transcription for 0, 4, 8, and 12 h. Total RNAs was extracted using RNAiso Plus TRIzol reagent (Takara, Japan, 9109) and analyzed by RT-qPCR. The mRNA expression for each group at the indicated times was calculated using the 2^-△△^Ct method and normalized to β-actin.

### RNA immunoprecipitation (RIP)

RIP assays were performed using the Magna RIP™ RNA-Binding Protein Immunoprecipitation Kit (Millipore, USA, 17-700), according to the manufacturer's instructions. First, cell lysates were prepared using cell lysis buffer (Beyotime, China, P0013) containing protease and RNase inhibitors. Next, 50 μl of magnetic beads were pre-incubated with either 5 μg of METTL3 (Proteintech, China, 15073-1-AP), YTHDF3 (Proteintech, China, 25537-1-AP), or IgG (Proteintech, China, 30000-0-AP) antibody for 30 min at room temperature. Subsequently, the antibody-bead mixture was added to the cell lysate and incubated at 4 °C for 4 hours on a rotator. Following this, the RNA-protein IP complexes were washed four times, after which a proteinase K digestion buffer was utilized for protein removal. In the final step, RNAs was purified using the phenol-chloroform method and prepared for qPCR analysis.

### MeRIP

M6A modifications of RNAs were measured using a methylated RNA immunoprecipitation (MeRIP) assay. Briefly, polyA+ RNA was purified using VAHTSTM mRNA Capture Beads (Vazyme, China, N401-01). Then RNAs were incubated with m6A antibody (Abcam, UK, ab151230) in 1ml IP buffer containing RNase inhibitor (Beyotime, China, R0102-2kU), 50 mM Tris-HCl, 750 mM NaCl, and 0.5% (v/v) Igepal CA-630 for 4 h at 4℃. After adequate washing, the immunoprecipitated mixture was digested using a high dose of proteinase K. The bound RNAs were then isolated using the phenol-chloroform method, followed by ethanol precipitation. These RNAs were subsequently used for the qPCR analysis.

### MDA assays

The MDA assay kit (Nanjing Jiancheng Bioengineering Institute, China, A003-4-1) was used according to the manufacturer's instructions. Briefly, 1×10^6^ cells were collected and lysed thoroughly using 100 μl of lysis buffer. 2 μl aliquot of the lysate was used to measure the protein concentration of the sample. The working mixtures were prepared using 100 μl samples and 1 ml working solution, then immersed in a 95℃-water bath for 40 min before quickly cooling under running water. The mixtures were centrifuged at 4000 rpm for 10 min. The 530 nm OD value of a 250 μl supernatant sample was measured using microplate reader (Thermofish, USA). Finally, the MDA content was calculated based on the manufacturer's instructions. The data were normalized to the control samples, as shown by the relative MDA level.

### GSH assays

The reduced GSH assay kit (Nanjing Jiancheng Bioengineering Institute, China, A006-2-1) was used according to the manufacturer's instructions. A total of 1×10^6^ cells were collected and thoroughly lysed in protein removal reagent M solution using the freeze-thaw method and subsequently centrifuged at 12000 rpm at 4℃ for 10 min. Afterward, 10 ul of the supernatant sample was added to a 96-well plate, followed by the addition of 150 ul of the detection working solution. The mixture was then incubated at room temperature in the dark for 5 min, followed by a further 25 min incubation after the addition of 50 ul NADPH solution. The absorbance at 412 nm was measured using a microplate reader, and the GSH content was calculated according to the manufacturer's instructions. The data were normalized to the control samples, as shown by the relative GSH level.

### Oil red O staining

After treatment, the cells were fixed with 4% polyformaldehyde for 10 min and then covered with a small amount of 60% isopropyl alcohol for 20 s. The cells were then stained with Oil Red O working solution (Servicebio, China, G1015-100ML) at room temperature in the dark for 30 min, followed by a quick wash with 60% isopropyl alcohol for 5 s, and then rinsed three times with pure water, with each rinse lasting 5 min. The images were captured using a Leica DMI8 microscope (Germany).

### ROS assay

The ROS assay kit (Beyotime, China, S0033S) was used according to the manufacturer's instructions. Briefly, the complete medium was removed from the cells, and then the cells were exposed to an appropriate volume of 10 μM DCFH-DA diluted in serum-free DMEM for 30 min in an incubator. Subsequently, 0.25% Trypsin was employed to collect the cells for flow cytometric analysis.

### C11-BODIPY

For lipid peroxidation assay, the C11-BODIPY 581/591 probe from Thermofisher (USA, D3861) was utilized following the manufacturer's instructions. In brief, the 5 μM C11-BODIPY 581/591 working solution was prepared in serum-free DMEM and incubated with cells for 30 min in the incubator. Ready-to-use DAPI staining solution (Beyotime, China, C1005) was used to stain the nuclei for 5 min. After washing twice, the cells were covered with HBSS, and the images of oxidized C11 (wavelength 505 -550 nm) were captured using a Leica DMI8 fluorescence microscope (Germany).

### Statistical analysis

All analyses were carried out using GraphPad Prism 10.1, with data presented as the mean ± SD. Comparisons between two groups were analyzed using Student's *t*-tests, whereas for multiple comparisons, a one-way ANOVA test was employed. Correlation analysis was performed using the Pearson's correlation method. All statistical analyses were two-sided. An estimate of variation was performed for each group of data. *:*P* < 0.05, **:*P* < 0.01, ***:*P* < 0.001, n.s.: *P* > 0.05

## Supplementary Material

Supplementary figures and tables.

## Abbreviations

HCC: hepatocellular carcinoma
TCGA: the cancer genome atlas
LIHC: liver hepatocellular carcinoma
CHOL: cholangio carcinoma
LIMORE: liver cancer model repository
LICOB: liver cancer organoid biobank
lncRNAs: long non-coding RNAs
CPC: coding potential calculator
OS: overall survival
DFS: disease-free survival
FISH: fluorescence *in situ* hybridization
3-MA: 3-methyladenine
ZVAD: Z-VAD-FMK
Fer-1: ferrostatin-1
MDA: malondialdehyde
ROS: reactive oxygen species
LPO: lipid peroxidation
GO: gene ontology
KEGG: kyoto encyclopedia of genes and genomes
GSEA: gene set enrichment analysis
RIP: RNA immunoprecipitation
MeRIP: methylated RNA immunoprecipitation
RT-qPCR: quantitative real-time PCR
IHC: immunohistochemistry
m6A: N6-methyladenosine
ActD: actinomycin D
