# Supplementary material for: Decreased lncRNA HNF4A-AS1 facilitates resistance to sorafenib-induced ferroptosis of hepatocellular carcinoma by reprogramming lipid metabolism
Source: Theranostics. 2024 Oct 21;14(18):7088–110. doi: 10.7150/thno.99197 (PMC11610135; doi:10.7150/thno.99197)
Supplement: Supplementary file 1 — Supplementary figures and tables. [file thnov14p7088s1.pdf]

Supplement figure:

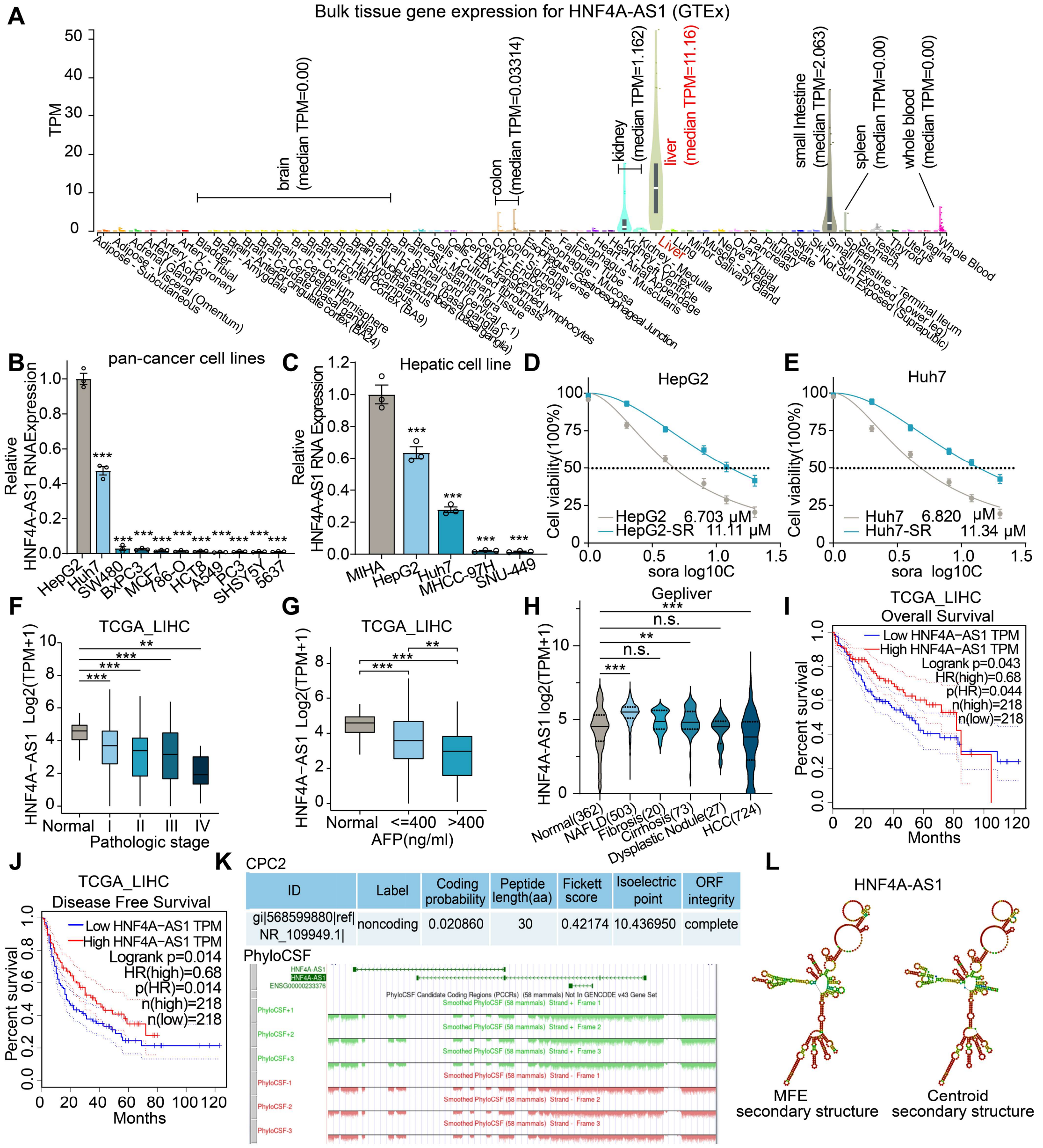

Fig. S1 HNF4A-AS1 is a liver-specific lncRNA and associated with HCC progression. (A) Analysis of HNF4A-AS1 expression in bulk tissues with GTEx database (https://www.gtexportal.org). (B) Expression levels of HNF4A-AS1 in cancer cells derived from different tissues. (C) HNF4A-AS1 expression in hepatic cell lines. (D-E) Sorafenib IC50 values in parental and resistant HepG2 and Huh7 cells. SR: sorafenib resistance. (F) Relationship between AFP expression and HNF4A-AS1 expression in TCGA LIHC database. (G) Expression levels of HNF4A-AS1 in different liver diseases according to the Gepliver website (http://www.gepliver.org). (H-I) Kaplan-Meier survival analysis, using the online tool GEPIA, assessing the overall

survival (OS) and disease-free survival (DFS) implications of HNF4A-AS1. (J) Expression levels of HNF4A-AS1 in different pathologic stages according to TCGA LIHC database. (K) The coding potential of HNF4A-AS1 was predicted using CPC2 (http://cpc2.gao-lab.org/) and PhyloCSF (https://github.com/mlin/PhyloCSF). (L)The secondary structure of HNF4A-AS1 was predicted using the RNAfold WebServer (http://rna.tbi.univie.ac.at/cgi-bin/RNAWebSuite/RNAfold.cgi).

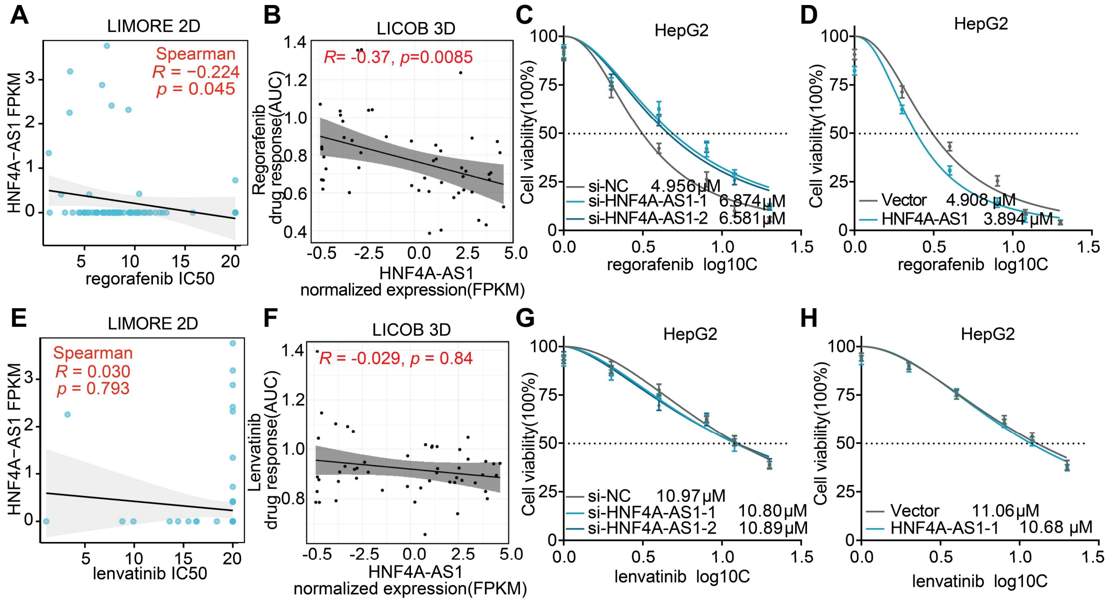

Fig. S2 The influence of HNF4A-AS1 on regorafenib and lenvatinib response. (A-B) The relationship between regorafenib response and HNF4A-AS1 expression was observed in primary liver cancer cells cultured in 2D (LIMORE, A) and in organoids cultured in 3D (LICOB, B) from the respective databases. Higher IC50 and AUC indicate lower sorafenib response. (C-D) Regorafenib IC50 values in HepG2 cells after knockdown or overexpression of HNF4A-AS1 level. (E-F) The relationship between lenvatinib response and HNF4A-AS1 expression was observed in primary liver cancer cells cultured in 2D (LIMORE, E) and in organoids cultured in 3D (LICOB, F) from the respective databases. Higher IC50 and AUC indicate lower sorafenib response. (G-H) Lenvatinib IC50 values in HepG2 cells after knockdown or overexpression of HNF4A-AS1 level.

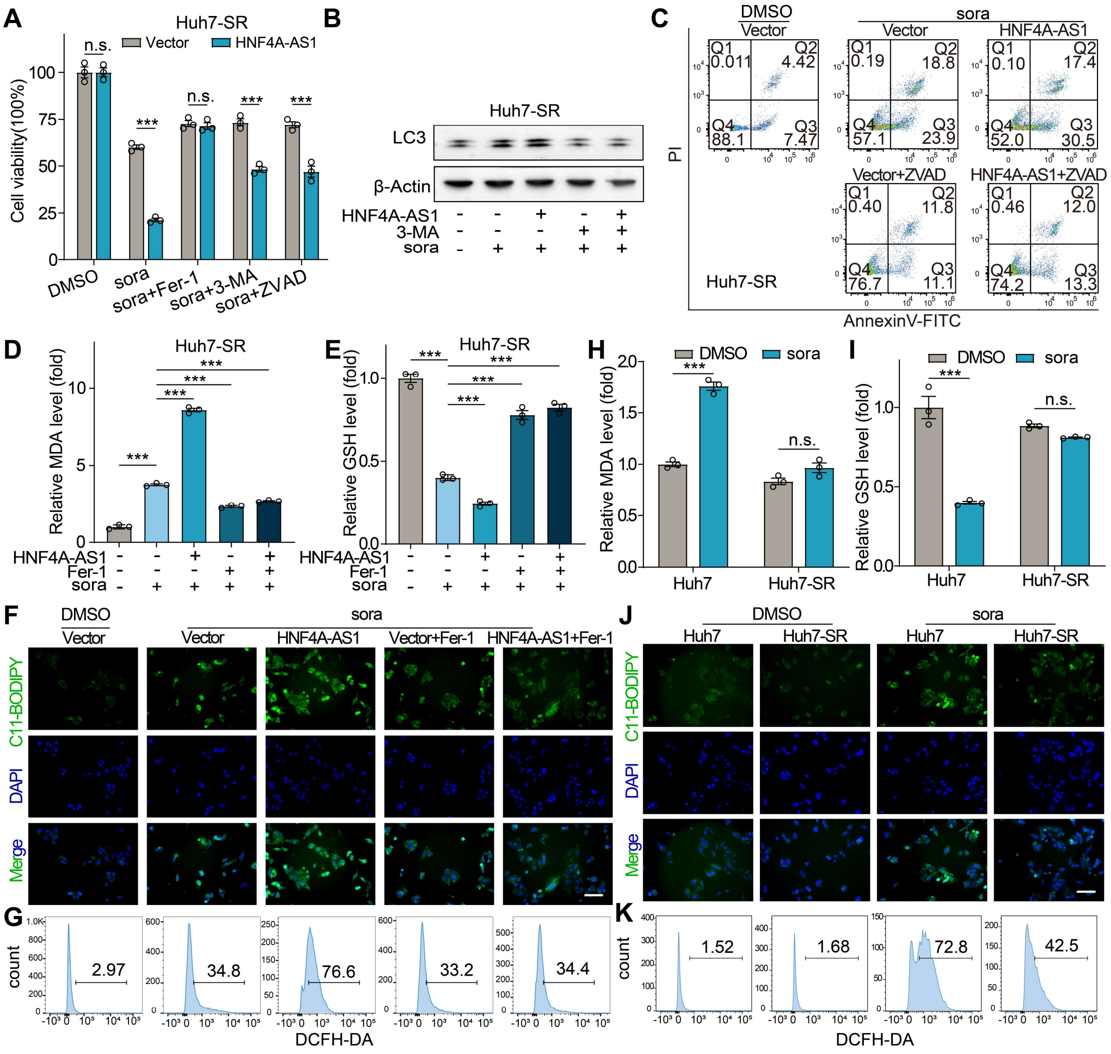

Fig. S3 HNF4A-AS1 regulates sorafenib response through ferroptosis. (A) Cell viability experiment assessing the effects of ferroptosis inhibitor ferrostatin-1 (Fer-1, 10 μM), autophagy inhibitor 3-methyladenine (3-MA, 10 μM) or apoptosis inhibitor Z-VAD-FMK (ZVAD, 50 μM) on cell viability after sorafenib treatment in Huh7-SR cells overexpressing HNF4A-AS1 or Vector. (B) Western blot analysis demonstrating the impact of HNF4A-AS1 overexpression and autophagy inhibitor 3-MA (10 μM) on autophagy in Huh7-SR cells after sorafenib treatment (10 μM). (C)AnnexinV/PI flow cytometry experiment showing the influence of HNF4A-AS1 overexpression and apoptosis inhibitor ZVAD (50 μM) on induced apoptosis in Huh7-SR cells after sorafenib treatment (10 μM). (D-G) MDA assay (D), GSH assay (E), C11-bodipy labeled LPO (F), and DCFH-DA labeled ROS (G) indicating the effects of HNF4A-AS1 overexpression and ferroptosis inhibitor Fer-1 on induced ferroptosis in Huh7-SR

cells after sorafenib treatment (10 μM). Scale bar:50 μm. (H-K) MDA assay (H), GSH assay (I), C11-bodipy labeled LPO (J), and DCFH-DA labeled ROS (K) demonstrating that ferroptosis is less prominent in the drug-resistant cell line Huh7-SR compared to the parental Huh7 cells under sorafenib treatment (10 μM). Scale bar:50 μm. Data shown are means ± SD from biological triplicates. ***:$P < 0.001$, n.s.:$P > 0.05$

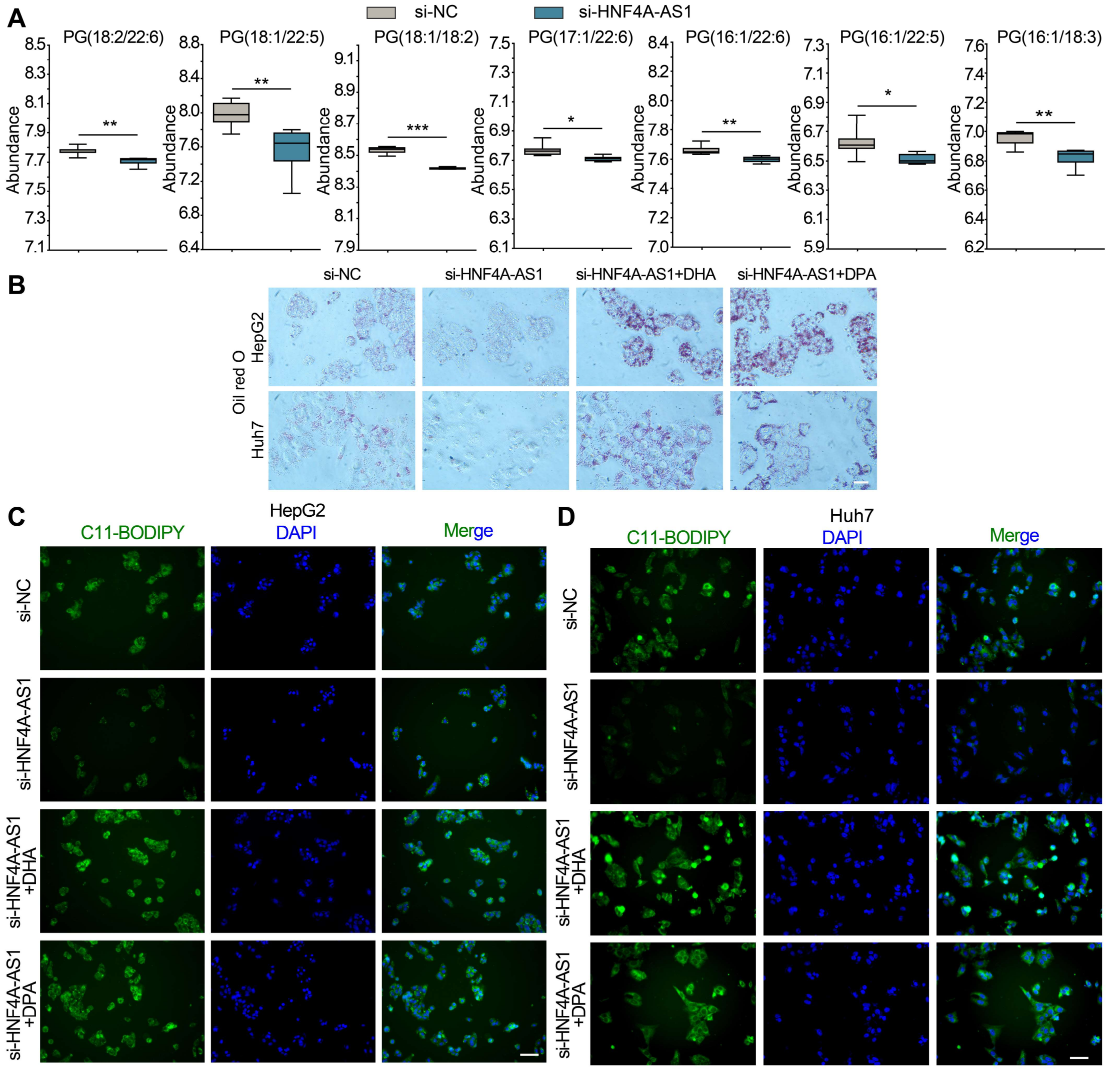

Fig. S4 Exogenous PUFA reverses the reduction in sorafenib-dependent ferroptosis caused by si-HNF4A-AS1. (A) Abundance of different phospholipids that contain PUFA are shown based on lipidomic in Figure 3A. (B) Oil red O staining of HepG2 and Huh7 cells transfected with si-NC or si-HNF4A-AS1 and treated with DHA or DPA (50 μM). Scale bar:10 μm. (C-D) C11-BODIPY staining of HepG2 and Huh7 cells transfected with si-NC or si-HNF4A-AS1 and treated with DHA or DPA (50 μM)

under sorafenib treatment (10 μM). Scale bar:50 μm. Data shown are means ± SD from 6 biological plicates. *:$P < 0.05$, **:$P < 0.01$, ***:$P < 0.001$

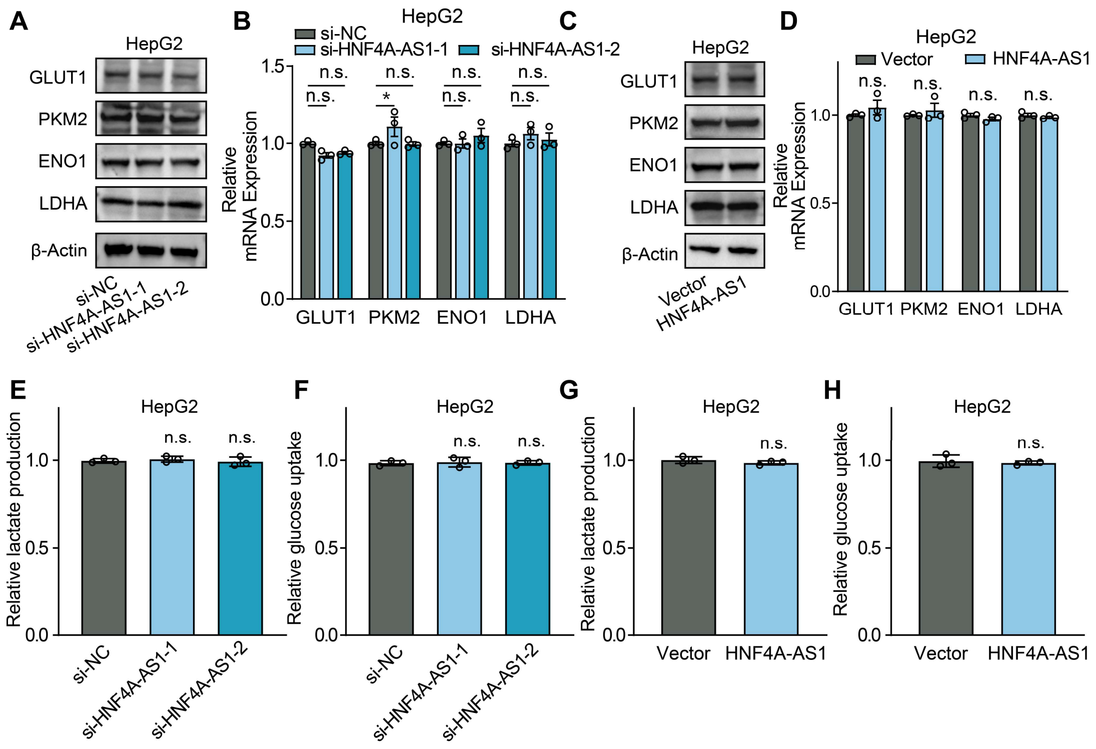

Fig. S5 HNF4A-AS1 have no influence on glycolysis. (A-B) The protein and RNA levels of glycolysis enzymes (GLUT1, PKM2, ENO1, LDHA) were detected after interference of HNF4A-AS1 by WB or qRT-PCR, respectively. (C-D) The protein and RNA levels of glycolysis enzymes (GLUT1, PKM2, ENO1, LDHA) were detected after overexpression of HNF4A-AS1 by WB or RT-PCR, respectively. (E-F) The levels of glucose uptake and lactate production in HepG2 transfected with si-NC or si-HNF4A-AS1 were measured. (G-H) Measurement of glucose uptake and lactate production levels in HepG2 transfected with HNF4A-AS1 overexpression. n.s.:$P > 0.05$

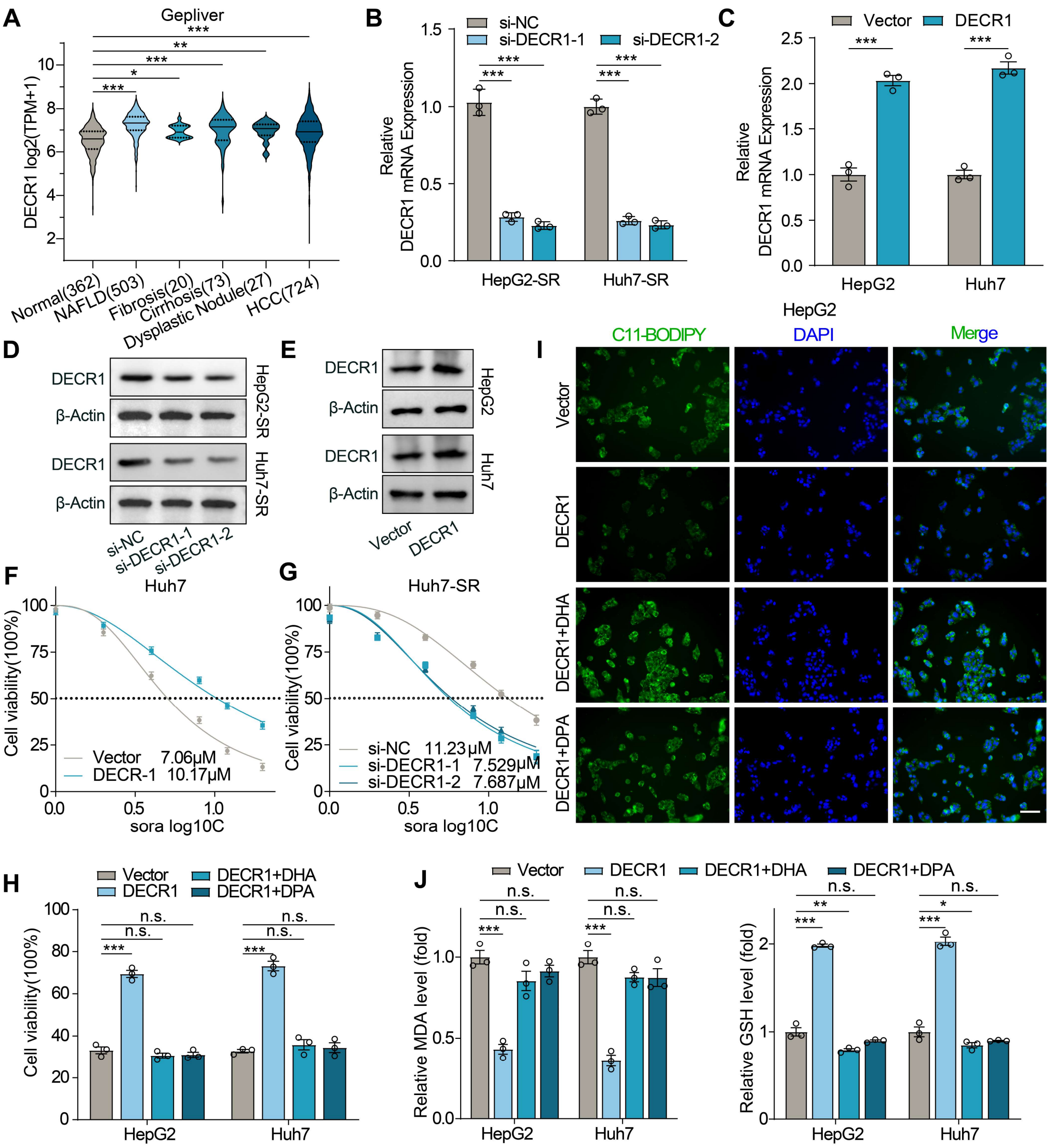

Fig. S6 HNF4A-AS1 mediates the tolerance of sorafenib-induced ferroptosis through DECR1. (A) Expression levels of DECR1 in different liver diseases from the Gepliver database. (B-E) RT-qPCR and western blot analysis of DECR1 expression in parental and resistant cells transfected with si-DECR1 or DECR1 overexpression plasmid respectively. (F-G) IC50 values of sorafenib were measured in Huh7 cells with overexpressing DECR1 and Huh7-SR cells with si-DECR1 interference. (H-J) Cell viability (H), LPO (I), MDA, and GSH (J) assays showed that DECR1 overexpression decreased ferroptosis under sorafenib treatment (10 μM) in HepG2 and Huh7 cells, and these effects were reversed by supplementing of DHA or DPA (50 μM). Scale bar:50 μm. Data shown are means ± SD from biological triplicates. *:$P < 0.05$, **:$P < 0.01$,

***:$P < 0.001$, n.s.:$P > 0.05$

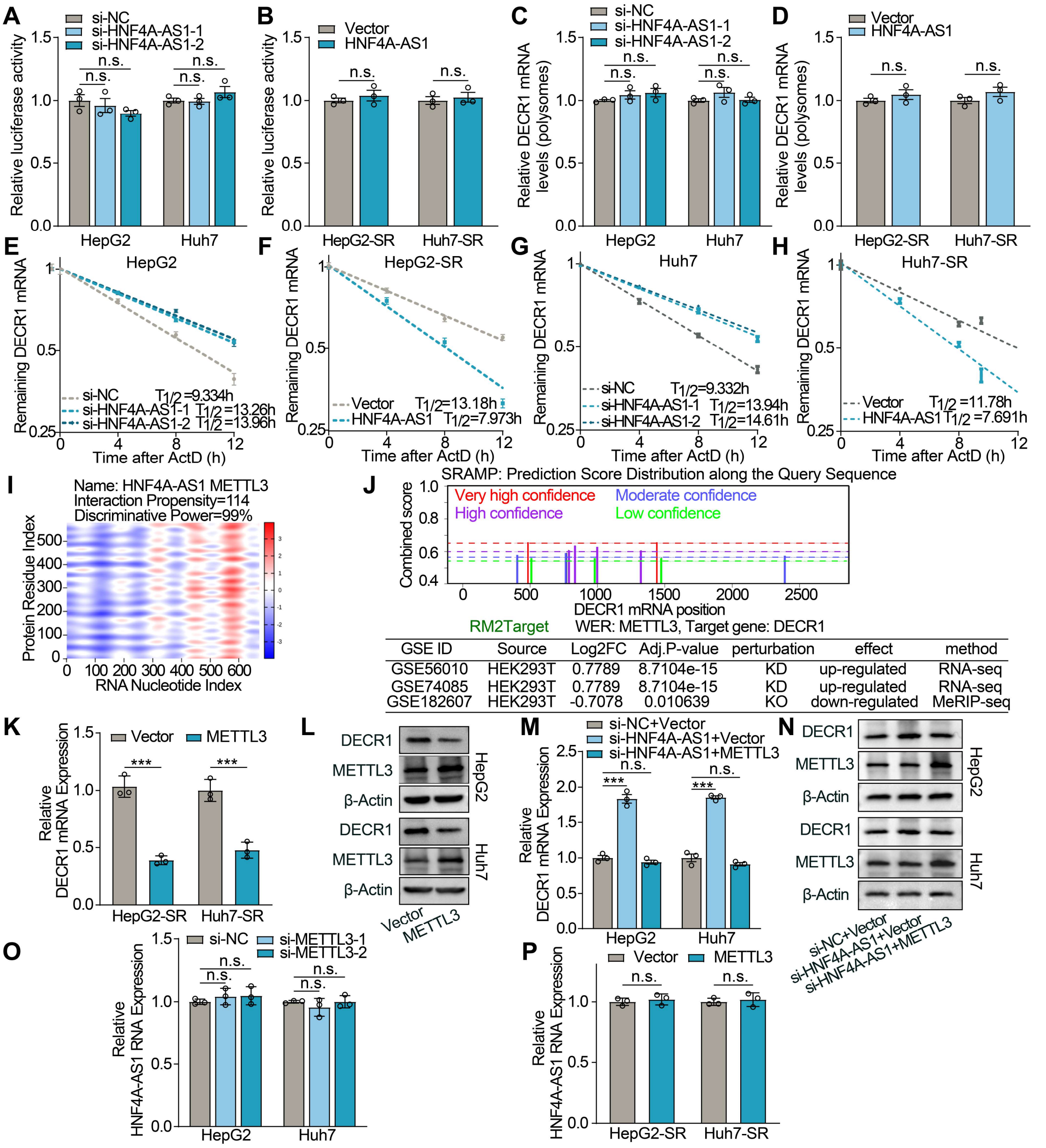

Fig. S7 METTL3-mediated m6A modification decreases DECR1 mRNA stability. (A-B) DECR1 promoter sequence luciferase reporter activity in parental and resistant cells respectively transfected with si-NC/si-HNF4A-AS1 or vector/HNF4A-AS1 were measured by luciferase reporter assay. Renilla luciferase served as a control. (C-D) The polysomes-conjunct DECR1 mRNA was measured in parental and resistant cells respectively transfected with si-NC/si-HNF4A-AS1 or vector/HNF4A-AS1. (E-H) RNA stability assay of DECR1 in parental and resistant cells respectively transfected with si-NC/si-HNF4A-AS1 or vector/HNF4A-AS1. (I) Heatmap showing the predicted

interaction between HNF4A-AS1 and METTL3 protein in catRAPID website (http://service.tartaglialab.com/page/catrapid_group). The colors represent the interaction scores ranging from -3 to 3 for individual amino acid and nucleotide pairs. The interaction propensity between HNF4A-AS1 and METTL3 protein is 114, and the discriminative power is 99%. (J) SRAMP (http://www.cuilab.cn/sramp) and RM2Target (http://rm2target.canceromics.org) databases suggested that DECR1 has two very high confident m6A modification sites and is negatively regulated by METTL3. (K-L) RT-qPCR and western blot analysis of DECR1 expression in HepG2-SR and Huh7-SR cells transfected with vector or METTL3 plasmid. (M-N) RT-qPCR and western blot analysis of DECR1 expression in HepG2 and Huh7 cells transfected with si-NC+vector, si-HNF4A-AS1+vector, or si-HNF4A-AS1+METTL3. (O-P) RT-qPCR analysis of HNF4A-AS1 expression in parental and resistant cells transfected with si-NC/si-METTL3 or vector/METTL3, respectively. Data shown are means ± SD from biological triplicates. ***: $P < 0.001$, n.s.: $P > 0.05$

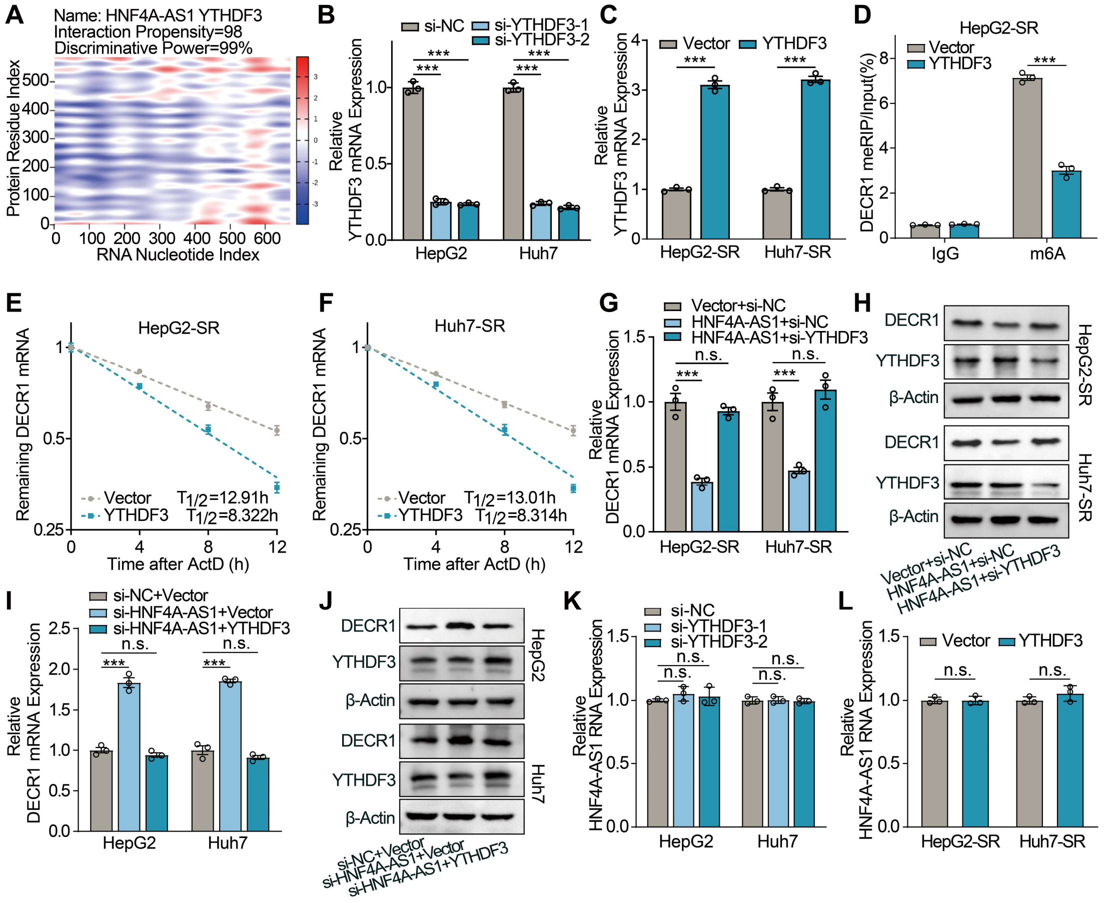

Fig. S8 YTHDF3-mediated m6A modification decreases DECR1 mRNA stability. (A) The heatmap shows the predicted interaction between HNF4A-AS1 and YTHDF3 protein in catRAPID website. The colors indicate the interaction scores (-3 to 3) of individual amino acid and nucleotide pairs. The interaction propensity between HNF4A-AS1 and YTHDF3 protein is 98, and the discriminative power is 99%. (B-C) RT-qPCR analysis to determine the knockdown efficiency of si-YTHDF3 and overexpression efficiency of YTHDF3 plasmid. (D) MeRIP-PCR of DECR1 mRNA using anti-m6A or anti-IgG antibodies in HepG2-SR cells transfected with vector or YTHDF3 plasmid. (E-F) RNA stability assay of DECR1 in HepG2 and Huh7 cells transfected with vector or YTHDF3 plasmid. (G-H) RT-qPCR and western blot analyses of DECR1 expression in HepG2-SR and Huh7-SR cells transfected with vector+si-NC, HNF4A-AS1+si-NC, or HNF4A-AS1+si-YTHDF3. (I-J) RT-qPCR and western blot analyses of DECR1 expression in HepG2 and Huh7 cells transfected with si-NC+vector, si-HNF4A-AS1+vector, or si-HNF4A-AS1+YTHDF3. (K-L) RT-qPCR analysis of

HNF4A-AS1 expression in parental and resistant cells transfected with si-NC/si-YTHDF3 or vector/YTHDF3, respectively. Data shown are means ± SD from biological triplicates. ***:$P < 0.001$, n.s.:$P > 0.05$

Supplement table S1. The unsaturated fatty acid metabolism geneset.

ABCD1	CYP2A6	ELOVL1	PLA2G10	HADHB
ABCD2	CYP2A7	ELOVL2	PLA2G2F	ACSL1
ABHD12	CYP2B6	ELOVL3	PLA2G3	HSD17B4
ABHD6	CYP2C18	ELOVL4	PLA2G4A	MIR1908
ACAA1	CYP2C19	ELOVL5	PLA2G4B	MIR6734
ACOT8	CYP2C8	ELOVL6	PLA2G4C	ACADS
ACOX1	CYP2C9	ELOVL7	PLA2G4F	ACADVL
AKR1B1	CYP2D6	EPHX1	PLAA	ACSM3
AKR1C1	CYP2D7	FAAH	PNPLA8	ACSM6
AKR1C2	CYP2E1	FAAH2	PRXL2B	ECHS1
AKR1C3	CYP2F1	FABP5	PTGDS	HADH
AKR1C4	CYP2G1P	FADS1	PTGES	
ALOX12	CYP2J2	FADS2	PTGES2	
ALOX12B	CYP2S1	FADS2B	PTGES3	
ALOX15	CYP2U1	FADS3	PTGIS	
ALOX15B	CYP4A11	GPX1	PTGR1	
ALOX5	CYP4A22	GPX4	PTGR2	
ALOXE3	CYP4F11	GSTA1	PTGS1	
ANXA1	CYP4F12	GSTM1	PTGS2	
ATP6V1B1	CYP4F2	GSTM2	SCD	
AVP	CYP4F3	GSTP1	SCD5	
AVPR1A	CYP4F8	HPGD	SCP2	
AWAT1	CYP4Z1	HPGDS	SIRT1	
CBR1	DAGLA	IL1B	TBXAS1	
CD74	DAGLB	MGLL	TNFRSF1A	
CES2	DECR1	MIF	ZADH2	
CYP1A1	DECR2	MIR132	ACADL	
CYP1A2	DEGS1	MIR204	ACADM	
CYP1B1	EDN1	MIR766	ECI1	
CYP2A13	EDN2	PIBF1	HADHA	

Supplement table S2. Mass spectrometry data of proteins and peptides pulled down by HNF4A-AS1.

Accession	Gene	Description	Mw(kDa)	Antisense	Sense	log2(S/A)	Diff Sig
P06702	S100A9	Protein S100-A9	13.242	19.6903	25.0275	5.3372	++
P12273	PIP	Prolactin-inducible protein	16.572	20.7392	25.7864	5.0472	++
H0YKU1	TMOD3	Tropomodulin-3 (Fragment)	20.79	19.7689	24.7565	4.9876	++
P68363	TUBA1B	Tubulin alpha-1B chain	50.151	21.3219	26.0554	4.7335	++
Q15125	EBP	3-beta-hydroxysteroid-Delta(8),Delta(7)-isomerase	26.352	21.0126	25.3177	4.3051	++
O75131	CPNE3	Copine-3	60.13	19.7227	23.9612	4.2385	++
C9JRC4	PPP1R7	Protein phosphatase 1 regulatory subunit 7 (Fragment)	21.156	19.7444	23.8515	4.1071	++
A0A024R7W5	YTHDF3	YTH domain family, member 3, isoform CRA_a	58.311	19.1089	22.9989	3.89	++
B8ZZ77	PPIL3	Peptidyl-prolyl cis-trans isomerase	17.696	20.9539	24.5495	3.5956	++
Q02318	CYP27A1	Sterol 26-hydroxylase, mitochondrial	60.234	19.8505	23.4193	3.5688	++
Q14966	ZNF638	Zinc finger protein 638	220.62	22.3	25.7692	3.4692	++
H7C0D5	YIPF4	Protein YIPF4 (Fragment)	6.447	19.5167	22.8942	3.3775	++
A8MU39	PPP5C	Serine/threonine-protein phosphatase	42.394	20.3448	23.5265	3.1817	++
P35914	HMGCL	Hydroxymethylglutaryl-CoA lyase, mitochondrial	34.36	19.7528	22.6537	2.9009	++
C9JLU1	POLR2H	DNA-directed RNA polymerases I, II, and III subunit RPABC3 (Fragment)	16.925	20.3628	23.1482	2.7854	++
Q5T760	SRSF11	Serine/arginine-rich-splicing factor 11 (Fragment)	42.316	20.0835	22.647	2.5635	++
Q9UBV8	PEF1	Peflin	30.381	20.2028	22.7267	2.5239	++

C9JAX1	FXN	Frataxin intermediate form	14.91	20.8835	23.3367	2.4532	++
F5H6D8	METTL3	N6-adenosine-methyltransferase catalytic subunit	26.266	20.2772	22.6744	2.3972	++
A0A3B3IRQ9	ADAR	Double-stranded RNA-specific adenosine deaminase (Fragment)	128.27	21.2421	23.6031	2.361	++
A0A087X208	AGRN	Agrin	202.29	19.4348	21.7218	2.287	++
Q5T6F2	UBAP2	Ubiquitin-associated protein 2	117.11	20.5315	22.8055	2.274	++
Q9H3Z4	DNAJC5	DnaJ homolog subfamily C member 5	22.149	20.09	22.1352	2.0452	++
G5E9W3	CPSF3	Cleavage and polyadenylation specific factor 3, 73kDa, isoform CRA_b	73.476	19.5591	21.5991	2.04	++
C9JXK9	LPP	Lipoma-preferred partner (Fragment)	26.706	20.7811	22.7636	1.9825	++
A0A5H1ZRR1	PQBP1	Polyglutamine-binding protein 1 (Fragment)	24.935	20.6489	22.388	1.7391	++
Q96EL3	MRPL53	39S ribosomal protein L53, mitochondrial	12.107	21.0884	22.69	1.6016	++
Q5SZC9	ABRACL	Costars family protein ABRACL	7.5164	20.9567	22.4634	1.5067	++
H0Y6T7	NCSTN	Nicastrin (Fragment)	30.045	20.7456	22.2291	1.4835	++
Q9NTJ5	SACM1L	Phosphatidylinositol-3-phosphatase SAC1	66.966	20.2932	21.6467	1.3535	++
A8K7Q2	HSPA8	Heat shock cognate 71 kDa protein	45.338	20.8728	22.1737	1.3009	++
O75663	TIPRL	TIP41-like protein	31.444	21.607	22.9018	1.2948	++
E7EPF2	*RBMS1*	*RNA-binding motif, single-stranded-interacting protein 1 (Fragment)*	*13.713*	*21.0836*	*22.3698*	*1.2862*	++
Q9BRX8	PRXL2A	Peroxiredoxin-like 2A	25.764	19.5826	24.7477	5.1651	++
B0YJC4	VIM	Vimentin	49.653	18.4818	21.2929	2.8111	++
O60927	PPP1R11	E3 ubiquitin-protein ligase PPP1R11	13.952	21.2606	23.7539	2.4933	++

Q53S08	RAB6D	Ras-related protein Rab-6D	28.242	22.9739	25.0987	2.1248	++
Q9NU22	MDN1	Midasin	632.81	23.618	25.6155	1.9975	++
Q02413	DSG1	Desmoglein-1	113.75	21.586	23.563	1.977	++
Q8WXE9	STON2	Stonin-2	101.16	23.556	25.4292	1.8732	++
Q9H0U4	RAB1B	Ras-related protein Rab-1B	22.171	23.4598	25.1345	1.6747	++
J3QSB7	MTAP	Purine nucleoside phosphorylase	26.777	23.8731	25.5139	1.6408	++
P81605	DCD	Dermcidin	11.284	26.5673	28.1586	1.5913	++
P16104	H2AX	Histone H2AX	15.144	22.7457	24.2915	1.5458	++
G5E9X5	MCCC1	Methylcrotonoyl-CoA carboxylase subunit alpha, mitochondrial	48.216	22.7851	24.3141	1.529	++
Q7L2H7	EIF3M	Eukaryotic translation initiation factor 3 subunit M	42.502	22.6427	24.1608	1.5181	++
Q6P587	FAHD1	Acylpyruvase FAHD1, mitochondrial	24.843	22.2846	23.7776	1.493	++
H3BN98		40S ribosomal protein S15a (Fragment)	27.168	20.6261	22.0977	1.4716	++
Q5T8U5	SURF4	Surfeit locus protein 4	21.127	22.7494	24.2052	1.4558	++
Q9Y3C6	PPIL1	Peptidyl-prolyl cis-trans isomerase-like 1	18.237	24.4611	25.9099	1.4488	++
D6RFH4	CYB5B	Cytochrome b5 type B	15.208	25.5118	26.946	1.4342	++
P68400	CSNK2A1	Casein kinase II subunit alpha	45.143	25.0707	26.465	1.3943	++
Q9HAB8	PPCS	Phosphopantothenate--cysteine ligase	34.005	20.7493	22.1423	1.393	++
O43598	DNPH1	2-deoxynucleoside 5-phosphate N-hydrolase 1	19.108	23.2334	24.6223	1.3889	++
J3QKW7	DRG2	Developmentally-regulated GTP-binding protein 2	26.624	21.915	23.2839	1.3689	++
P43897	TSFM	Elongation factor Ts, mitochondrial	35.39	22.0053	23.37	1.3647	++

Q08554	DSC1	Desmocollin-1	99.986	20.5567	21.9177	1.361	++
P62854	RPS26	40S ribosomal protein S26	13.015	25.7329	27.0919	1.359	++
G3V1K2	RXYLT1	Ribitol-5-phosphate xylosyltransferase 1	21.208	24.5348	25.8467	1.3119	++
Q86VF7	NRAP	Nebulin-related-anchoring protein	197.07	23.133	24.4389	1.3059	++
Q9BX68	HINT2	Histidine triad nucleotide-binding protein 2, mitochondrial	17.162	23.6558	24.9456	1.2898	++
B4DJM9	SARS2	Serine--tRNA ligase, mitochondrial	26.313	18.5831	19.8389	1.2558	++
Q07666	KHDRBS1	KH domain-containing, RNA-binding, signal transduction-associated protein 1	48.227	25.9475	27.1827	1.2352	++
P53597	SUCLG1	Succinate--CoA ligase [ADP/GDP-forming] subunit alpha, mitochondrial	36.249	23.5648	24.7999	1.2351	++
P62314	SNRPD1	Small nuclear ribonucleoprotein Sm D1	13.281	24.1125	25.2957	1.1832	++
P21964	COMT	Catechol O--methyltransferase	30.037	21.5358	22.7037	1.1679	++
Q8WWC4	MAIP1	m-AAA protease-interacting protein 1, mitochondrial	32.544	21.9324	23.0854	1.153	+
F2Z2Y6	LSM8	U6 snRNA-associated Sm-like protein LSm8	7.3713	22.4022	23.5452	1.143	+
A0A096LNJ1	GATD3B	Glutamine amidotransferase-like class 1 domain-containing protein 3B, mitochondrial (Fragment)	11.004	22.2905	23.4162	1.1257	+
H0YL19	WDR61	WD repeat-containing protein 61	23.241	21.1351	22.2443	1.1092	+
C9JA28	SSR3	Signal sequence receptor subunit gamma	20.161	23.7256	24.8143	1.0887	+
H0YMM5	DUT	Deoxyuridine 5-triphosphate nucleotidohydrolase	14.306	25.2587	26.3449	1.0862	+

Q14344	GNA13	Guanine nucleotide-binding protein subunit alpha-13	44.049	22.1854	23.251	1.0656	+
O15067	PFAS	Phosphoribosylformylglycinamidine synthase	144.73	23.5652	24.6183	1.0531	+
Q9NP97	DYNLRB1	Dynein light chain roadblock-type 1	10.921	26.4087	27.4176	1.0089	+
Q5JRG1	NUP58	Nucleoporin p58/p45 (Fragment)	49.095	21.4335	22.4413	1.0078	+

Supplement table S3. The sequence of siRNA

SiRNA Targets	Sequences (5’- 3’)
HNF4A-AS1#1	Sense:GGAGCUGGGAUCUGACACUTT
	Antisense: AGUGUCAGAUCCCAGCUCCTT
HNF4A-AS1#2	Sense: CCUGGGCAGAAGCUUUAAATT
	Antisense: UUUAAAGCUUCUGCCCAGGTT
METTL3#1	Sense: CCUGCAAGUAUGUUCACUATT
	Antisense: UAGUGAACAUACUUGCAGGTT
METTL3#2	Sense: GCUACCUGGACGUCAGUAUTT
	Antisense: AUACUGACGUCCAGGUAGCTT
YTHDF3#1	Sense: UCCAUCCAUUGGAUUUCCAUAUUTT
	Antisense: AAUAUGGAAAUCCAAUGGAUGGATT
YTHDF3#2	Sense: GACCUAAAGGGCAAGGAAAUAAATT
	Antisense: UUUAUUUCCUUGCCCUUUAGGUCTT
DECR1#1	Sense: AUGCUACCACCUAAUAGUUTT
	Antisense: AACUAUUAGGUGGUAGCAUCG
DECR1#2	Sense: AGAAGAACUCGCAAAUCUUTT
	Antisense: AAGAUUUGCGAGUUCUUCUAC

Supplement table S4. Primers of RT-qPCR

Gene	Sequence (5' to 3')
HNF4A-AS1	F: TCCTTTAGCGGCTTCCAAGG
	R: GCCTGTCTTCAAGTCACCGA

HNF4A	F: TGCGACTCTCCAAAACCCTC
	R: ATTGCCCATCGTCAACACCT
DECR1	F: TCTTCAAAAAGCGATGCTACCA
	R: CTATCACGCACTGAGCACCT
SCP2	F: ACTGATTTGCCAAGCTCGTTT
	R: TCAGGCCAGATTTCTCATAGCA
ALOX5	F: CTCAAGCAACACCGACGTAAA
	R: CCTTGTGGCATTTGGCATCG
ELOVL5	F: ATGGTTTGTCGTCAGTCCCTT
	R: AGCTGGTCTGGATGATTGTCA
ACSL1	F: CGACGAGCCCTTGGTGTATTT
	R: GGTTTCCGAGAGCCTAAACAA
GLUT1	F: ATTGGCTCCGGTATCGTCAAC
	R: GCTCAGATAGGACATCCAGGGTA
ENO1	F: TGGTGTCTATCGAAGATCCCTT
	R: CCTTGGCGATCCTCTTTGG
PKM2	F: ATAACGCCTACATGGAAAAGTGT
	R: TAAGCCCATCATCCACGTAGA
LDHA	F: TTGACCTACGTGGCTTGGAAG
	R: GGTAACGGAATCGGGCTGAAT
GAPDH	F: CTGGGCTACACTGAGCACC
	R: AAGTGGTCGTTGAGGGCAATG
ACTB	F: CATGTACGTTGCTATCCAGG
	R: CTCCTTAATGTCACGCACGAT
U6	F: CTCGCTTCGGCAGCACAT
	R: TTTGCGTGTCATCCTTGCG
METTL3	F: TTGTCTCCAACCTTCCGTAGT
	R: CCAGATCAGAGAGGTGGTGTAG

YTHDF3	F: TCAGAGTAACAGCTATCCACCA<br>R: GGTTGTCAGATATGGCATAGGCT

Supplement table S5. Antibodies used for western blotting.

Antibody	Company	Catalog number	Dilution
DECR1	Abcam	Ab198848	1:1000
METTL3	Proteintech	15073-1-AP	1:1000
YTHDF3	Proteintech	25537-1-AP	1:1000
β-actin	Proteintech	66009-1-Ig	1:3000
GLUT1	Proteintech	21829-1-AP	1:1000
PKM2	Proteintech	60268-1-Ig	1:1000
ENO1	Proteintech	11204-1-AP	1:1000
LDHA	Proteintech	19987-1-AP	1:1000
HRP-conjugated Affinipure Goat Anti-Rabbit IgG(H+L)	Proteintech	SA00001-2	1:3000
HRP-conjugated Affinipure Goat Anti-Mouse IgG(H+L)	Proteintech	SA00001-1	1:3000
